# Supplementary material for: Effects of polyethylene glycol 2 L alone or with ascorbic acid compared with polyethylene glycol 4 L alone for bowel preparation before colonoscopy: protocol for a systematic review and network meta-analysis
Source: BMJ Open. 2017 Oct 16;7(10):e018217. doi: 10.1136/bmjopen-2017-018217 (PMC5652536; doi:10.1136/bmjopen-2017-018217)
Supplement: Supplementary file 2 [file bmjopen-2017-018217supp002.pdf]

<table>
<tr><th colspan="5">Basic Characteristics of Included into Study</th></tr>
<tr><td colspan="2" rowspan="2">Study Information</td><td>Author</td><td>Year</td><td>Country</td></tr>
<tr><td></td><td></td><td></td></tr>
<tr><td rowspan="4">Methods</td><td>Allocation</td><td colspan="3"></td></tr>
<tr><td>Duration</td><td colspan="3"></td></tr>
<tr><td>Blinding</td><td colspan="3"></td></tr>
<tr><td>Location</td><td colspan="3"></td></tr>
<tr><td rowspan="9">Participants</td><td>Diagnosis</td><td colspan="3">Pts were diagnosed as GC based histology.</td></tr>
<tr><td rowspan="2">Age</td><td>Study Group</td><td colspan="2">Control Group</td></tr>
<tr><td></td><td colspan="2"></td></tr>
<tr><td rowspan="2">Sex</td><td>Study Group</td><td colspan="2">Control Group</td></tr>
<tr><td></td><td colspan="2"></td></tr>
<tr><td rowspan="2">Length of Illness</td><td>Study Group</td><td colspan="2">Control Group</td></tr>
<tr><td></td><td colspan="2"></td></tr>
<tr><td>Inclusion criteria</td><td colspan="3"></td></tr>
<tr><td>Exclusion criteria</td><td colspan="3"></td></tr>
<tr><td rowspan="2">Interventions</td><td>Treatment Group</td><td>Content</td><td colspan="2"></td></tr>
<tr><td>Control group</td><td>Content</td><td colspan="2"></td></tr>
<tr><td>Outcomes</td><td colspan="4"></td></tr>
<tr><td>Notes</td><td colspan="4"></td></tr>
</table>

Drop-outs	Drop out due to	Study group	Control group
	the numbers of patients in the early stage		
	the numbers of patients in the late stage		

Binary Data			
Outcomes	Name of Outcome	Event number	Total number
	Study group		
	Control group		

Binary Data			
Outcomes	Name of Outcome	Event number	Total number
	Study group		
	Control group		

Binary Data			
Outcomes	Name of Outcome	Event number	Total number
	Study group		
	Control group		

Continuous Data				
Outcomes	Name of outcome	Data extraction		
		Median	Range	P
	ECT group			
	Paroxetin group			

Continuous Data				
Outcomes	Name of outcome	Data extraction		
		Median	Range	P
	ECT group			
	Paroxetin group			

Assessing of Risk of Bias Tool		
**Item**	**Description**	**Risk of Bias**
**Sequence Generation**	Describe the method used to generate the allocation sequence in sufficient detail to allow an assessment of whether it should produce comparable groups	Was the allocation sequence adequately generated?
	Comment:	Low Risk
**Allocation Concealment**	Describe the method used to conceal the allocation sequence in sufficient detail to determine whether intervention allocations could have been foreseen	Was the allocation adequately concealed?
	Comment:	Low Risk
**Blinding of Participants and Personnel**	Describe all measures used, if any, to blind study participants and personnel from knowledge of which intervention a participant received. Provide any information relating to whether the intended blinding was effective	Was knowledge of the allocated intervention adequately prevented during the study?
	Comment:	Low Risk
**Blinding of Outcome Assessors**	Describe all measures used, if any, to blind outcome assessors from knowledge of which intervention a participant received. Provide any information relating to whether the intended blinding was effective.	Was knowledge of the allocated intervention adequately prevented during the study?
	Comment:	Unclear
**Incomplete Outcome Data**	Describe the completeness of outcome data for each main outcome, including attrition and exclusions from the analysis. State whether attrition and exclusions were reported, the numbers in each intervention group (compared with total randomized participants), reasons for attrition/exclusions where reported, and	Were incomplete outcome data adequately addressed?
	Comment:	Low Risk
**Selective Outcome Reporting**	State how the possibility of selective outcome reporting was examined by the review authors and what was found.	Are reports of the study free of suggestion of selective
	Comment:	Low Risk
**Other Bias**	State any important concerns about bias not addressed in the other domains in the tool. If particular questions/entries were re-specified in the review protocol, responses should be provided for each question/entry	Was the study apparently free of other problems that could put it at high risk of bias?
	Comment:	Low Risk
