# Supplementary material for: Effects of polyethylene glycol 2 L alone or with ascorbic acid compared with polyethylene glycol 4 L alone for bowel preparation before colonoscopy: protocol for a systematic review and network meta-analysis
Source: BMJ Open. 2017 Oct 16;7(10):e018217. doi: 10.1136/bmjopen-2017-018217 (PMC5652536; doi:10.1136/bmjopen-2017-018217)
Supplement: Author's manuscript [file bmjopen-2017-018217.draft_revisions.pdf]

BMJ Open

BMJ Open is committed to open peer review. As part of this commitment we make the peer review history of every article we publish publicly available.

When an article is published we post the peer reviewers' comments and the authors' responses online. We also post the versions of the paper that were used during peer review. These are the versions that the peer review comments apply to.

The versions of the paper that follow are the versions that were submitted during the peer review process. They are not the versions of record or the final published versions. They should not be cited or distributed as the published version of this manuscript.

BMJ Open is an open access journal and the full, final, typeset and author-corrected version of record of the manuscript is available on our site with no access controls, subscription charges or pay-per-view fees (http://bmjopen.bmj.com).

If you have any questions on BMJ Open's open peer review process please email editorial.bmjopen@bmj.com

BMJ Open

Comparative efficacy of different PEG-based regimes for bowel preparation prior to colonoscopy: protocol for a systematic review and network meta-analysis

Journal:	*BMJ Open*
Manuscript ID	bmjopen-2017-018217
Article Type:	Protocol
Date Submitted by the Author:	13-Jun-2017
Complete List of Authors:	Tian, Xu; Chongqing Cancer Hospital, Department of Gastroenterology<br>Chen, Wei-Qing; Chongqing Cancer Hospital, Department of Gastroenterology<br>Huang, Jie-Li; Chongqing Cancer Hospital, Department of Gastroenterology<br>He, Lan-Ying; Chongqing Cancer Hospital, Department of Gastroenterology<br>Liu, Bang-Lun; Chongqing Cancer Hospital, Department of Gastroenterology<br>Liu, Xi; Chongqing Cancer Hospital, Department of Gastroenterology<br>Zhou, Hang; Chongqing Cancer Hospital, Department of Gastroenterology<br>Liu, Bing-Rong; Chongqing Cancer Hospital, Department of Gastroenterology
**Primary Subject Heading**:	Evidence based practice
Secondary Subject Heading:	Gastroenterology and hepatology, Evidence based practice, Nursing, Oncology
Keywords:	Colonoscopy, Bowel preparation, Polyethylene glycol, Ascorbic acid, Meta-analysis

SCHOLARONE™ Manuscripts

Comparative efficacy of different PEG-based regimes for bowel preparation prior to colonoscopy: protocol for a systematic review and network meta-analysis

Xu Tian[1*], Wei-Qing Chen[1*], Jie-Li Huang[1], Lan-Ying He[1], Bang-Lun Liu[1], Xi Liu[1], Hang Zhou[1], Bing-Rong Liu[2]

*These authors have contributed equally to this work as first author.

[1]Department of Gastroenterology, Chongqing Cancer Institute & Hospital & Cancer Center, Chongqing 400030, China

[2]Hospital of Digestive Diseases, the First Affiliated Hospital of Zhengzhou University, Zhengzhou 450052, China

Corresponding author: Wei-Qing Chen, MD, Department of Gastroenterology, Chongqing Cancer Institute & Hospital & Cancer Center, 181 Hanyu Road, Shapingba District, Chongqing 400030, China; telephone number: (86) 0 18222275023, e-mail: chenwq20140809@163.com

For peer review only

ABSTRACT

Introduction: Colonoscopy has been regarded as a standard method of early detecting and removing gastrointestinal lesions, while adequate bowel preparation is the prerequisite of determining the diagnostic accuracy and treatment safety of this process. PEG-based bowel preparation regime remains the first recommendation, but optimal option is still uncertainty. The aim of this systematic review and network meta-analysis of randomized controlled trials (RCTs) is to determine the optimal PEG-based bowel preparation regime before colonoscopy.

Methods and analysis: We will assign two investigators to independently search all potential citations, screen records, abstract essential information, and appraise risk of bias accordingly. And then, random effects pairwise and network meta-analyses of RCTs comparing all available PEG-based bowel preparation regimes with each other will be performed by using RevMan 5.3 (Copenhagen: The Nordic Cochrane Centre, The Cochrane Collaboration, 2013), Stata 14 (StataCorp, Texas) and WinBUGS 1.4 (imperial College School of Medicine at St Mary's, London) from January 2000 to April 2017. The surface under the cumulative ranking curve (SCURA) will also be calculated in order to rank regimes.

Ethics and dissemination: The ethics approval and patient written informed consent will not be required because of all analyses in the present study will be performed based on data from published studies. We will submit our systematic review and network meta-analysis to a peer-reviewed scientific journal for publication.

For peer review only - http://bmjopen.bmj.com/site/about/guidelines.xhtml

For peer review only

BACKGROUND

Colorectal cancer (CRC) is one of the most common cancers diagnosed in the world and it is also the major contributor to cancer-associated morbidity and mortality [1]. Colonoscopy has been considered as the most effective method for early detection and prevention of CRC [2]. Published evidences suggested that early detection and endoscopic resection of polyps and abnormal lesions gastrointestinal tract can reduce approximately 50% mortality of CRC [3, 4]. It is noted that, however, the adequate bowel preparation is the prerequisite of guaranteeing diagnostic accuracy and therapeutic safety of colonoscopy [5]. Issued data illustrated a sever fact that more than 40% of colonoscopy failures resulted from inadequate bowel preparation [6]. Moreover, inadequate bowel preparation also caused other negative consequences such as missed detection of polyps or lesions, increased risk of procedure-related complications, and increased economic costs [7]. Several factors can affect the quality of bowel preparation [8], and low patient-based compliance, poor palatability of bowel preparation solution, and inevitable requirement of drinking a large volume of preparation solution account for 20% to 25% inadequate bowel preparations [7]. However, low patient-based compliance with recommend regime play a decisive role in the overall success of the procedure [9].

For the purpose of improving quality of bowel preparation, several regimes have been developed such as polyethylene glycol (PEG)-based solutions, sodium phosphate (NaP), and sodium picosulfate solutions. Of these all regimes, PEG-based regimes are still first recommendation [10]. Several modified regimes, including split-dose regime,

For peer review only - http://bmjopen.bmj.com/site/about/guidelines.xhtml

low-volume regime, low-volume plus ascorbic acid (Asc), have been designed because patients are difficult to intake traditional 4L PEG owing to the large volume of fluid and poor palatability [11]. A series of randomized controlled trials (RCTs) have been performed to investigate the comparative efficacy of split dose versus single dose [12], low volume (2L) plus Asc versus traditional volume (4L) [13], and low volume plus Asc versus low volume [14]. However, the study regarding low volume versus traditional volume, low volume versus low volume plus Asc with split dose, and low volume versus traditional volume with split dose have not yet been identified. Moreover, individual study is difficult to identify subtle clinical differences owing to the smaller patient number [15]. Several meta-analyses have also been performed to evaluate the efficacy of low volume versus traditional volume [16], low volume versus plus Asc versus traditional volume [17], and split dose versus single dose [18, 19]. Traditional meta-analysis methods, however, is unable to investigate the comparative efficacy of more than 2 interventions.

In order to solve the limitations of traditional meta-analysis technique, Bayesian network meta-analysis based on Markov Chain Monte Carlo (MCMC) and Gibbs Sampling, the expansion of pairwise meta-analysis, has been developed to evaluate the comparative efficacy of multiple treatments which are not directly compared in individual RCT [20]. And thus, we proposed this network meta-analysis to establish the optimal PEG-based bowel preparation regime prior to colonoscopy. We designed this systematic review and network meta-analysis on May 10, 2017; and we expected to finish this study on September 30, 2017.

For peer review only - http://bmjopen.bmj.com/site/about/guidelines.xhtml

METHODS AND DESIGN

We designed and completed this protocol for systematic review and network meta-analysis according to the preferred reporting items for systematic review and meta-analysis protocols (PRISMA-P) 2015: elaboration and explanation [21]. We will perform this traditional pairwise and network meta-analysis in accordance with the Cochrane Handbook for Systematic Reviews of Interventions [22] and report all results according to the preferred reporting items for systematic review and meta-analysis for network meta-analysis (PRISMA-NMA) [23].

Selection criteria

In our meta-analysis, the study will be considered if the following inclusion criteria are met: (i) Patients: all adult patients undergoing elective colonoscopy, irrespective of outpatients and inpatients; (ii) Intervention: all PEG-based bowel preparation regimes including 4L PEG and 2L PEG plus Asc with single or split dose and did not combine with other drugs, we drawn the possible evidence network according to the targeted regimes in terms of bowel preparation efficacy (see **Figure 1**); (iii) Outcomes: bowel preparation efficacy (BPE) was regarded as primary outcome, and the secondary outcomes including compliance with recommend regime (CP), preference to repeat the same regime (PRSR), acceptance to regime (AT), and adverse events (AEs); (iv) Study design: only RCTs were included, abstract with sufficient data was also considered.

Study will be excluded if it met at least one of following criteria: (i) essential

For peer review only - http://bmjopen.bmj.com/site/about/guidelines.xhtml

information cannot be extracted; (ii) duplication with poor methodology and insufficient data; and (iii) non-original research types such as review, editorial, letter to the editor and comments.

Definition of outcomes

In our systemic review and network meta-analysis, the BPE was also regarded as successful bowel preparation, and it was defined an Ottawa score of < 5, or an excellent or good bowel preparation designation on the Aronchik scale or other non-validated 3-, 4-, or 5-point scales (excellent, good, fair, poor, very poor). CP was defined as adherence to the bowel preparation prescribed or consumption of at least 75% of the prescribed bowel preparation. PRSR, AT and AEs were measured by using the specified questionnaires in each eligible study (i.e. defined by individual study).

Identification of citations

We will firstly electronically search the PubMed, Cochrane Central Register of Controlled Trials (CENTRAL), and EMBASE in order to capture all potential records investigating the comparative efficacy of different PEG-based bowel preparation regimes from January 2000 to April 2017. "*Colonoscopy*", "*polyethylene glycols*", and "*random*" were used to construct search algorithms in accordance with the requests of targeted databases, and all possible search algorithms have been documented in **Table 1**.

After electronic search, we will also hand check the reference lists of all eligible

For peer review only - http://bmjopen.bmj.com/site/about/guidelines.xhtml

studies and topic-related review and electronically retrieve the Clinicaltrial.gov for the purpose of covering all potential eligible study. It is noted that, however, only studies published in English will be considered in our systematic review and network meta-analysis.

Data extraction

We have designed a standard data extraction form before performed our previous two systematic reviews and network meta-analyses. We will consequently assign two reviewers to abstract the basic information and data for specific outcome from eligible study, such as first author, publication year, age of participants, sample size, bowel preparation regimes, and outcomes of interest using this standard data extraction form [24]. We will contact the corresponding author if sufficient data of a eligible study cannot be abstracted from full-text. We set the consensus principle to be the method of resolving divergences between reviewers.

Quality assessment of individual study

We will assign two independent reviewers to appraise the risk of bias from seven domains including randomization sequence generation, allocation concealment, blinding of participants, blinding of study personnel, blinding of outcome assessors, incomplete outcome data, selective reporting and other bias with the Cochrane risk of bias assessment tool [22, 25]. A study will be assigned into a level of '*high risk of bias*', '*unclear risk of bias*' or '*low risk of bias*' according to the match level between actual

For peer review only - http://bmjopen.bmj.com/site/about/guidelines.xhtml

information and evaluation criteria [22].

Statistical analysis

We will firstly perform traditional pairwise meta-analysis based on random effect model, which incorporates within- and between-studies heterogeneity, to estimate the summarized odd ratio (OR) and 95% confidence intervals (CIs) [26]. Chi^2 method will be adopted to test the heterogeneity [27] and I^2 statistic will be used to estimate the proportion of the overall variation that is attributable to between-study heterogeneity [28]. The value of I^2 statistic is larger than 50% indicating substantial heterogeneity [28]. We will draw the funnel plot to identify publication bias if the number of studies analyzed was more than 10 [29]. The studies with more than two comparison groups will be quantitatively incorporated in pairwise meta-analysis according to the specific comparison.

Following the traditional pairwise meta-analysis, random-effects network meta-analysis will be performed according to the methods described by Chaimani and colleagues [30]. The initial values, automatically generated from software, will be used to fit the model [31]. We plan to perform 70000 iterations and 30000 burn-in for each outcome and convergence.

We will generate the comparison-adjusted funnel plot to assess the small-study effects when the number of studies included in one pair of comparison was more than 10 [32]. We will calculate the inconsistency factor based on the loop-specific method to assess the inconsistency [30].

For peer review only - http://bmjopen.bmj.com/site/about/guidelines.xhtml

The surface under the cumulative ranking curve (SUCRA) will also be drawn in order to rank all PEG-based bowel preparation regimes and a higher value suggests better results for respective regime [33].

All analyses will be conducted by using the RevMan 5.3 (Copenhagen: The Nordic Cochrane Centre, The Cochrane Collaboration, 2013), Stata 14 (StataCorp, Texas) and WinBUGS 1.4 (imperial College School of Medicine at St Mary's, London).

DISCUSSION

The CRC is one of the most common malignancies, and issued statistics illustrated that it is the fourth contributor to the cancer-death worldwide [1]. Colonoscopy has been regarded as the standard process of early preventing and detecting CRC in clinical practice [2]. However, diagnostic accuracy and operation safety during performing colonoscopy mainly depend on the quality of bowel preparation [34]. Although several novel bowel preparation regimes have been developed in order to improve the tolerability and compliance of patients, the PEG-based regimes have been first-line recommendation [10]. It is noted that several modified regimes have been applied in clinical practice, but no primary study and traditional pairwise meta-analysis comparing various PEG-based bowel preparation regimes with each other has been published. And thus it is still debate which PEG-based regime should be optimally described. We hence proposed this network meta-analysis to determine the optimal PEG-based regime for the purpose of facilitating the informed-decision making.

This network meta-analysis was one of the first to compare the direct and indirect

For peer review only - http://bmjopen.bmj.com/site/about/guidelines.xhtml

effects of different PEG-based regimes for bowel preparation prior to colonoscopy. The results of the present network meta-analysis will influence evidence-based decision-making in bowel preparation regime prescription, since it will be fundamental for reliable recommendations in the consideration of bowel preparation regime before colonoscopy.

STRENGTHS AND LIMITATIONS OF THIS STUDY

The protocol addresses the important question of which PEG-based bowel preparation regime offers the most benefits for bowel preparation efficacy before colonoscopy.

The present network meta-analysis has a clearly established aim, stringent inclusion criteria, state-of-the-art methods for data collection and quantitative synthesis.

Limitations include variations in administration times of drinking the same bowel preparation regimes, diet description prior to colonoscopy, type of colonoscopies, and assessment tool for bowel preparation efficacy.

ACKNOWLEDGMENTS

We would like to appreciate the editor and anonymous referees for their helps.

AUTHORS CONTRIBUTIONS

X.T. and W.-Q.C. conceived and designed this study; X.T. and J.-L.H. searched and selected studies; L.-Y.H. and B.-L.L. extracted essential information; X.T. and B.-R.L. assessed the risk of bias; X.T., W.-Q.C., X.L. and H.Z. performed statistical analyses;

For peer review only - http://bmjopen.bmj.com/site/about/guidelines.xhtml

X.T. and W.-Q.C. interpreted the pooled results; X.T., W.-Q.C. and B.-R.L. drafted the manuscript; all authors approved this manuscript to be considered for publication.

FUNDING STATEMENT

This research received no specific grant from any funding agency in the public, commercial or not-for-profit sectors.

COMPETING INTERESTS STATEMENT

The authors report no declarations of interest.

For peer review only

For peer review only - http://bmjopen.bmj.com/site/about/guidelines.xhtml

REFERENCES

1. Torre LA, Bray F, Siegel RL, Ferlay J, Lortet-Tieulent J and Jemal A. Global cancer statistics, 2012. CA Cancer J Clin. 2015; 65(2):87-108.

2. Lieberman DA, Weiss DG, Bond JH, Ahnen DJ, Garewal H and Chejfec G. Use of colonoscopy to screen asymptomatic adults for colorectal cancer. Veterans Affairs Cooperative Study Group 380. The New England journal of medicine. 2000; 343(3):162-168.

3. Quintero E, Castells A, Bujanda L, Cubiella J, Salas D, Lanas A, Andreu M, Carballo F, Morillas JD, Hernandez C, Jover R, Montalvo I, Arenas J, Laredo E, Hernandez V, Iglesias F, et al. Colonoscopy versus fecal immunochemical testing in colorectal-cancer screening. The New England journal of medicine. 2012; 366(8):697-706.

4. Zauber AG, Winawer SJ, O'Brien MJ, Lansdorp-Vogelaar I, van Ballegooijen M, Hankey BF, Shi W, Bond JH, Schapiro M, Panish JF, Stewart ET and Waye JD. Colonoscopic polypectomy and long-term prevention of colorectal-cancer deaths. The New England journal of medicine. 2012; 366(8):687-696.

5. Marmo R, Rotondano G, Riccio G, Marone A, Bianco MA, Stroppa I, Caruso A, Pandolfo N, Sansone S, Gregorio E, D'Alvano G, Procaccio N, Capo P, Marmo C and Cipolletta L. Effective bowel cleansing before colonoscopy: a randomized study of split-dosage versus non-split dosage regimens of high-volume versus low-volume polyethylene glycol solutions. Gastrointestinal endoscopy. 2010; 72(2):313-320.

6. Ponchon T, Boustiere C, Heresbach D, Hagege H, Tarrerias AL and Halphen M. A

For peer review only - http://bmjopen.bmj.com/site/about/guidelines.xhtml

low-volume polyethylene glycol plus ascorbate solution for bowel cleansing prior to colonoscopy: the NORMO randomised clinical trial. Dig Liver Dis. 2013; 45(10):820-826.

7. Froehlich F, Wietlisbach V, Gonvers JJ, Burnand B and Vader JP. Impact of colonic cleansing on quality and diagnostic yield of colonoscopy: the European Panel of Appropriateness of Gastrointestinal Endoscopy European multicenter study. Gastrointestinal endoscopy. 2005; 61(3):378-384.

8. Kaminski MF, Regula J, Kraszewska E, Polkowski M, Wojciechowska U, Didkowska J, Zwierko M, Rupinski M, Nowacki MP and Butruk E. Quality indicators for colonoscopy and the risk of interval cancer. The New England journal of medicine. 2010; 362(19):1795-1803.

9. Ness RM, Manam R, Hoen H and Chalasani N. Predictors of inadequate bowel preparation for colonoscopy. Am J Gastroenterol. 2001; 96(6):1797-1802.

10. Mathus-Vliegen E, Pellise M, Heresbach D, Fischbach W, Dixon T, Belsey J, Parente F, Rio-Tinto R, Brown A, Toth E, Crosta C, Layer P, Epstein O and Boustiere C. Consensus guidelines for the use of bowel preparation prior to colonic diagnostic procedures: colonoscopy and small bowel video capsule endoscopy. Current medical research and opinion. 2013; 29(8):931-945.

11. Ell C, Fischbach W, Bronisch HJ, Dertinger S, Layer P, Runzi M, Schneider T, Kachel G, Gruger J, Kollinger M, Nagell W, Goerg KJ, Wanitschke R and Gruss HJ. Randomized trial of low-volume PEG solution versus standard PEG + electrolytes for bowel cleansing before colonoscopy. Am J Gastroenterol. 2008; 103(4):883-893.

For peer review only

For peer review only - http://bmjopen.bmj.com/site/about/guidelines.xhtml

12. Kim M, Park J, Park J, Kim H, Jang H, Joo H, Kim J, Choi J, Heo N, Park S, Kim T and Yang S. (2017). Does Polyethylene Glycol (PEG) Plus Ascorbic Acid Induce More Mucosal Injuries than Split-Dose 4-L PEG during Bowel Preparation? Gut and liver, pp. 237-243.

13. Rivas J, Perez A, Hernandez M, Schneider A and Castro F. (2014). Efficacy of morning-only 4 liter sulfa free polyethylene glycol vs 2 liter polyethylene glycol with ascorbic acid for afternoon colonoscopy. World journal of gastroenterology, pp. 10620-10627.

14. Kanie H, Kojima I, Araki H, Kusakabe A, Shimizu S, Yamada T, Hayashi K and Nakazawa T. A randomized clinical trial of low-volume polyethylene glycol versus low-volume polyethylene glycol with ascorbic acid solutions. Gastrointestinal endoscopy. 2016; 83(5):AB226.

15. Wang J, Meng XH, Guo ZM, Wu YH and Zhao JG. Interventions for treating displaced midshaft clavicular fractures: a Bayesian network meta-analysis of randomized controlled trials. Medicine (Baltimore). 2015; 94(11):e595.

16. Godfrey J, Clark R, Choudhary A, Ashraf I, Matteson M and Bechtold M. Low-volume polyethylene glycol and ascorbic acid for bowel preparation prior to colonoscopy: A meta-analysis. American Journal of Gastroenterology. 2011; 106:S527.

17. Xie Q, Chen L, Zhao F, Zhou X, Huang P, Zhang L, Zhou D, Wei J, Wang W and Zheng S. A meta-analysis of randomized controlled trials of low-volume polyethylene glycol plus ascorbic acid versus standard-volume polyethylene glycol solution as bowel preparations for colonoscopy. PloS one. 2014; 9(6):e99092.

For peer review only

For peer review only - http://bmjopen.bmj.com/site/about/guidelines.xhtml

18. Avalos DJ, Sarkis FS, Keihanian T, Berry AC, Castro- Pavia F, O'Rourke C, Sultan S and Sussman DA. Same-day morning bowel regimens vs. Split regimens for colonoscopy: A meta-analysis of randomized controlled trials. Gastroenterology. 2016; 150(4):S839.

19. Ménard C, Barkun AN, Martel M, Restellini S and Kherad O. Split-dose versus same-day bowel preparations for colonoscopy: A meta-analysis. Gastroenterology. 2016; 150(4):S838.

20. Mills EJ, Ioannidis JP, Thorlund K, Schunemann HJ, Puhan MA and Guyatt GH. How to use an article reporting a multiple treatment comparison meta-analysis. Jama. 2012; 308(12):1246-1253.

21. Shamseer L, Moher D, Clarke M, Ghersi D, Liberati A, Petticrew M, Shekelle P, Stewart LA and Group P-P. Preferred reporting items for systematic review and meta-analysis protocols (PRISMA-P) 2015: elaboration and explanation. BMJ. 2015; 349:g7647.

22. Higgins JP and Green S. Cochrane Handbook For Systematic Reviews Of Interventions Version 5.0.0. Naunyn-Schmiedebergs Archiv für experimentelle Pathologie und Pharmakologie. 2009; 210(2):S38.

23. Hutton B, Salanti G, Caldwell DM, Chaimani A, Schmid CH, Cameron C, Ioannidis JP, Straus S, Thorlund K, Jansen JP, Mulrow C, Catala-Lopez F, Gotzsche PC, Dickersin K, Boutron I, Altman DG, et al. The PRISMA extension statement for reporting of systematic reviews incorporating network meta-analyses of health care interventions: checklist and explanations. Ann Intern Med. 2015; 162(11):777-784.

For peer review only - http://bmjopen.bmj.com/site/about/guidelines.xhtml

24. Song GM, Tian X, Zhang L, Ou YX, Yi LJ, Shuai T, Zhou JG, Zeng Z and Yang HL. Immunonutrition Support for Patients Undergoing Surgery for Gastrointestinal Malignancy: Preoperative, Postoperative, or Perioperative? A Bayesian Network Meta-Analysis of Randomized Controlled Trials. Medicine (Baltimore). 2015; 94(29):e1225.

25. Zeng X, Zhang Y, Kwong JS, Zhang C, Li S, Sun F, Niu Y and Du L. The methodological quality assessment tools for preclinical and clinical studies, systematic review and meta-analysis, and clinical practice guideline: a systematic review. J Evid Based Med. 2015; 8(1):2-10.

26. DerSimonian R and Laird N. Meta-analysis in clinical trials. Controlled clinical trials. 1986; 7(3):177-188.

27. Bowden J, Tierney JF, Copas AJ and Burdett S. Quantifying, displaying and accounting for heterogeneity in the meta-analysis of RCTs using standard and generalised Q statistics. BMC medical research methodology. 2011; 11:41.

28. Higgins JPT and Thompson SG. Quantifying heterogeneity in a meta-analysis. Statistics in Medicine. 2002; 21(11):1539-1558.

29. Tian X, Zhou JG, Zeng Z, T. S, Yi LJ, Ma L, Wang Y, Cao H and Song GM. Cetuximab in patients with esophageal cancer: a systematic review and meta-analysis of randomized controlled trials. Medical Oncology. 2015; 32(4):1-10.

30. Chaimani A, Higgins JPT, Mavridis D, Spyridonos P and Salanti G. Graphical Tools for Network Meta-Analysis in STATA. Plos One. 2013; 8(10):e76654-e76654.

31. Sutton DA, Ades AE, Cooper N and Abrams K. Use of Indirect and Mixed

For peer review only

For peer review only - http://bmjopen.bmj.com/site/about/guidelines.xhtml

Treatment Comparisons for Technology Assessment. Pharmacoeconomics. 2008; 26(9):753-767.

32. Chen LX, Zhou ZR, Li YL, Ning GZ, Zhang TS, Zhang D and Feng SQ. Comparison of Bone Mineral Density in Lumbar Spine and Fracture Rate among Eight Drugs in Treatments of Osteoporosis in Men: A Network Meta-Analysis. Plos One. 2015; 10(5).

33. Georgia S, Ades AE and Ioannidis JPA. Graphical methods and numerical summaries for presenting results from multiple-treatment meta-analysis: an overview and tutorial. Journal of Clinical Epidemiology. 2011; 64(2):163-171.

34. Rapier R and Houston C. A prospective study to assess the efficacy and patient tolerance of three bowel preparations for colonoscopy. Gastroenterol Nurs. 2006; 29(4):305-308.

For peer review only

For peer review only - http://bmjopen.bmj.com/site/about/guidelines.xhtml

FIGURE LENGENDS

Figure 1 Possible evidence network of all possible PEG-based bowel preparation regimes in terms of bowel preparation efficacy. The yellow solid line indicates direct comparisons between regimes which were directly compared in original studies. The brown node represents the each PEG-based bowel preparation regime. PEG = polyethylene glycol, SD = split dose, Asc = ascorbic acid.

For peer review only

For peer review only - http://bmjopen.bmj.com/site/about/guidelines.xhtml

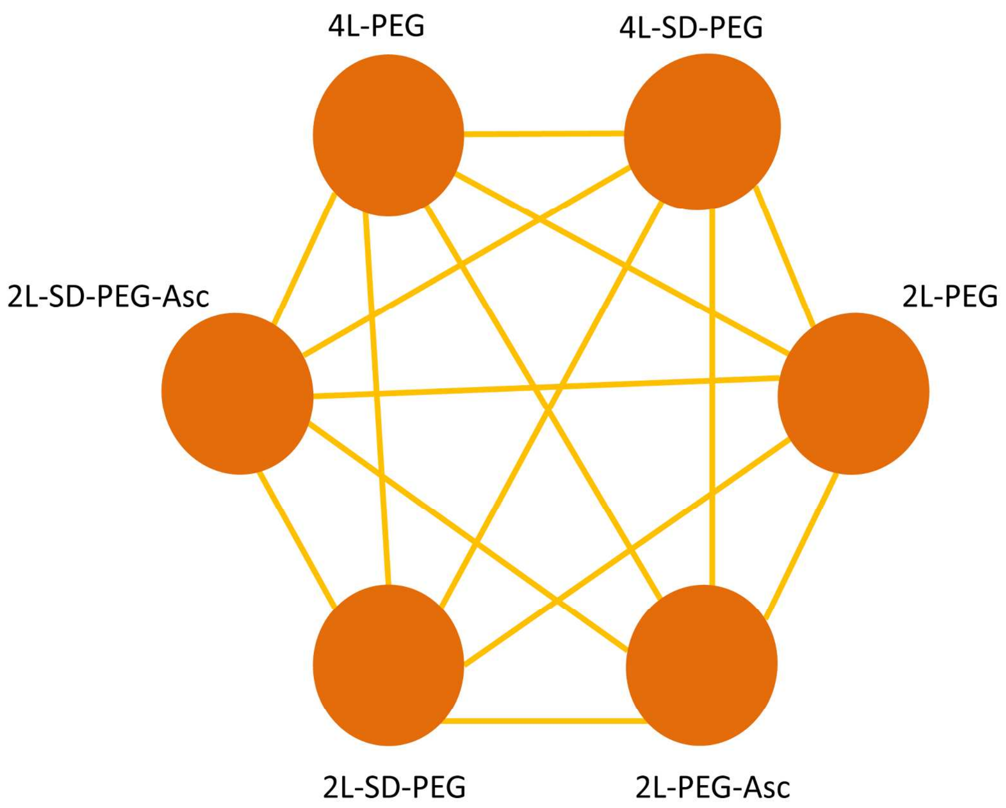

The yellow solid line indicates direct comparisons between regimes which were directly compared in original studies. The brown node represents the each PEG-based bowel preparation regime. PEG = polyethylene glycol, SD = split dose, Asc = ascorbic acid.

185x148mm (300 x 300 DPI)

CENTRAL Search Algorithm

ID	Search
#1	Polyethylene Glycol*:ti,ab,kw or Macrogol*:ti,ab,kw or Glycol, Polyethylene:ti,ab,kw or Glycols, Polyethylene:ti,ab,kw or Polyethylene Oxide*:ti,ab,kw (Word variations have been searched)
#2	Oxide, Polyethylene:ti,ab,kw or Oxides, Polyethylene:ti,ab,kw or Polyethyleneoxide*:ti,ab,kw or Polyoxyethylene*:ti,ab,kw or Tritons:ti,ab,kw (Word variations have been searched)
#3	MeSH descriptor: [Polyethylene Glycols] explode all trees
#4	#1 or #2 or #3
#5	Colonoscop*:ti,ab,kw or Colonoscopic Surgical Procedure*:ti,ab,kw or Procedure, Colonoscopic Surgical:ti,ab,kw or Procedures, Colonoscopic Surgical:ti,ab,kw or Surgical Procedure, Colonoscopic:ti,ab,kw (Word variations have been searched)
#6	Surgery, Colonoscopic:ti,ab,kw or Surgical Procedures, Colonoscopic:ti,ab,kw or Colonoscopic Surger*:ti,ab,kw or Surgeries, Colonoscopic:ti,ab,kw (Word variations have been searched)
#7	MeSH descriptor: [Colonoscopy] explode all trees
#8	#5 or #6 or #7
#9	random*:ti,ab,kw (Word variations have been searched)
#10	MeSH descriptor: [Randomized Controlled Trial] explode all trees
#11	MeSH descriptor: [Randomized Controlled Trials as Topic] explode all trees
#12	#9 or #10 or #11
#13	#4 and #8 and #12

For peer review only

PubMed Search Algorithm

Search	Query
#8	Search (((("Polyethylene Glycols"[Mesh]) OR ((((((((((Polyethylene Glycol*[Title/Abstract]) OR Macrogol*[Title/Abstract]) OR Glycol, Polyethylene[Title/Abstract]) OR Glycols, Polyethylene[Title/Abstract]) OR Polyethylene Oxide*[Title/Abstract]) OR Oxide, Polyethylene[Title/Abstract]) OR Oxides, Polyethylene[Title/Abstract]) OR Polyethyleneoxide*[Title/Abstract]) OR Polyoxyethylene*[Title/Abstract]) OR Tritons[Title/Abstract]))) AND (("Colonoscopy"[Mesh]) OR (((((((((Colonoscop*[Title/Abstract]) OR Colonoscopic Surgical Procedure*[Title/Abstract]) OR Procedure, Colonoscopic Surgical[Title/Abstract]) OR Procedures, Colonoscopic Surgical[Title/Abstract]) OR Surgical Procedure, Colonoscopic[Title/Abstract]) OR Surgery, Colonoscopic[Title/Abstract]) OR Surgical Procedures, Colonoscopic[Title/Abstract]) OR Colonoscopic Surger*[Title/Abstract]) OR Surgeries, Colonoscopic[Title/Abstract]))) AND random*[Title/Abstract] Sort by: PublicationDate
#7	Search random*[Title/Abstract] Sort by: PublicationDate
#6	Search ("Colonoscopy"[Mesh]) OR (((((((((Colonoscop*[Title/Abstract]) OR Colonoscopic Surgical Procedure*[Title/Abstract]) OR Procedure, Colonoscopic Surgical[Title/Abstract]) OR Procedures, Colonoscopic Surgical[Title/Abstract]) OR Surgical Procedure, Colonoscopic[Title/Abstract]) OR Surgery, Colonoscopic[Title/Abstract]) OR Surgical Procedures, Colonoscopic[Title/Abstract]) OR Colonoscopic Surger*[Title/Abstract]) OR Surgeries, Colonoscopic[Title/Abstract]) Sort by: PublicationDate
#5	Search ((((((((Colonoscop*[Title/Abstract]) OR Colonoscopic Surgical Procedure*[Title/Abstract]) OR Procedure, Colonoscopic Surgical[Title/Abstract]) OR Procedures, Colonoscopic Surgical[Title/Abstract]) OR Surgical Procedure, Colonoscopic[Title/Abstract]) OR Surgery, Colonoscopic[Title/Abstract]) OR Surgical Procedures, Colonoscopic[Title/Abstract]) OR Colonoscopic Surger*[Title/Abstract]) OR Surgeries, Colonoscopic[Title/Abstract] Sort by: PublicationDate
#4	Search "Colonoscopy"[Mesh] Sort by: PublicationDate
#3	Search ("Polyethylene Glycols"[Mesh]) OR ((((((((((Polyethylene Glycol*[Title/Abstract]) OR Macrogol*[Title/Abstract]) OR Glycol, Polyethylene[Title/Abstract]) OR Glycols, Polyethylene[Title/Abstract]) OR Polyethylene Oxide*[Title/Abstract]) OR Oxide, Polyethylene[Title/Abstract]) OR Oxides, Polyethylene[Title/Abstract]) OR Polyethyleneoxide*[Title/Abstract]) OR Polyoxyethylene*[Title/Abstract]) OR Tritons[Title/Abstract]) Sort by: PublicationDate
#2	Search (((((((((Polyethylene Glycol*[Title/Abstract]) OR Macrogol*[Title/Abstract]) OR Glycol, Polyethylene[Title/Abstract]) OR Glycols, Polyethylene[Title/Abstract]) OR Polyethylene Oxide*[Title/Abstract]) OR Oxide, Polyethylene[Title/Abstract]) OR Oxides, Polyethylene[Title/Abstract]) OR Polyethyleneoxide*[Title/Abstract]) OR Polyoxyethylene*[Title/Abstract]) OR Tritons[Title/Abstract] Sort by: PublicationDate
#1	Search "Polyethylene Glycols"[Mesh] Sort by: PublicationDate

For peer review only

Embase Search Algorithm

No. Query

#10. polyethylene:ab,ti AND glycol*:ab,ti OR macrogol*:ab,ti OR (glycol,:ab,ti AND polyethylene:ab,ti) OR (glycols,:ab,ti AND polyethylene:ab,ti) OR (polyethylene:ab,ti AND oxide*:ab,ti) OR (oxide,:ab,ti AND polyethylene:ab,ti) OR (oxides,:ab,ti AND polyethylene:ab,ti) OR polyethyleneoxide*:ab,ti OR polyoxyethylene:ab,ti OR tritons:ab,ti OR 'macrogol derivative'/exp AND (colonoscop*:ab,ti OR (colonoscopic:ab,ti AND surgical:ab,ti AND procedure*:ab,ti) OR (procedure,:ab,ti AND colonoscopic:ab,ti AND surgical:ab,ti) OR (procedures,:ab,ti AND colonoscopic:ab,ti AND surgical:ab,ti) OR (surgical:ab,ti AND procedure,:ab,ti AND colonoscopic:ab,ti) OR (surgery,:ab,ti AND colonoscopic:ab,ti) OR (surgical:ab,ti AND procedures,:ab,ti AND colonoscopic:ab,ti) OR (colonoscopic:ab,ti AND surger*:ab,ti) OR (surgeries,:ab,ti AND colonoscopic:ab,ti) OR 'colonoscopy'/exp) AND (random*:ab,ti OR 'randomized controlled trial'/exp OR 'randomized controlled trial (topic)'/exp)

#9. random*:ab,ti OR 'randomized controlled trial'/exp OR 'randomized controlled trial (topic)'/exp

#8. 'randomized controlled trial'/exp OR 'randomized controlled trial (topic)'/exp

#7. random*:ab,ti

#6. colonoscop*:ab,ti OR (colonoscopic:ab,ti AND surgical:ab,ti AND procedure*:ab,ti) OR (procedure,:ab,ti AND colonoscopic:ab,ti AND surgical:ab,ti) OR (procedures,:ab,ti AND colonoscopic:ab,ti AND surgical:ab,ti) OR (surgical:ab,ti AND procedure,:ab,ti AND colonoscopic:ab,ti) OR (surgery,:ab,ti AND colonoscopic:ab,ti) OR (surgical:ab,ti AND procedures,:ab,ti AND colonoscopic:ab,ti) OR (colonoscopic:ab,ti AND surger*:ab,ti) OR (surgeries,:ab,ti AND colonoscopic:ab,ti) OR 'colonoscopy'/exp

#5. 'colonoscopy'/exp

#4. colonoscop*:ab,ti OR (colonoscopic:ab,ti AND surgical:ab,ti AND procedure*:ab,ti) OR (procedure,:ab,ti AND colonoscopic:ab,ti AND surgical:ab,ti) OR (procedures,:ab,ti AND colonoscopic:ab,ti AND surgical:ab,ti) OR (surgical:ab,ti AND procedure,:ab,ti AND colonoscopic:ab,ti) OR (surgery,:ab,ti AND colonoscopic:ab,ti) OR (surgical:ab,ti AND

For peer review only

procedures,:ab,ti AND colonoscopic:ab,ti) OR (colonoscopic:ab,ti AND surger*:ab,ti) OR (surgeries,:ab,ti AND colonoscopic:ab,ti)

#3. polyethylene:ab,ti AND glycol*:ab,ti OR macrogol*:ab,ti OR (glycol,:ab,ti AND polyethylene:ab,ti) OR (glycols,:ab,ti AND polyethylene:ab,ti) OR (polyethylene:ab,ti AND oxide*:ab,ti) OR (oxide,:ab,ti AND polyethylene:ab,ti) OR (oxides,:ab,ti AND polyethylene:ab,ti) OR polyethyleneoxide*:ab,ti OR polyoxyethylene:ab,ti OR tritons:ab,ti OR 'macrogol derivative'/exp

#2. 'macrogol derivative'/exp

#1. polyethylene:ab,ti AND glycol*:ab,ti OR macrogol*:ab,ti OR (glycol,:ab,ti AND polyethylene:ab,ti) OR (glycols,:ab,ti AND polyethylene:ab,ti) OR (polyethylene:ab,ti AND oxide*:ab,ti) OR (oxide,:ab,ti AND polyethylene:ab,ti) OR (oxides,:ab,ti AND polyethylene:ab,ti) OR polyethyleneoxide*:ab,ti OR polyoxyethylene:ab,ti OR tritons:ab,ti

For peer review only

PRISMA-P (Preferred Reporting Items for Systematic review and Meta-Analysis Protocols) 2015 checklist: recommended items to address in a systematic review protocol*

Section and topic	Item No	Checklist item	Page
**ADMINISTRATIVE INFORMATION**			
Title:			
Identification	1a	Identify the report as a protocol of a systematic review	1
Update	1b	If the protocol is for an update of a previous systematic review, identify as such	n.a.
Registration	2	If registered, provide the name of the registry (such as PROSPERO) and registration number	n.a.
Authors:			
Contact	3a	Provide name, institutional affiliation, e-mail address of all protocol authors; provide physical mailing address of corresponding author	1
Contributions	3b	Describe contributions of protocol authors and identify the guarantor of the review	10,11
Amendments	4	If the protocol represents an amendment of a previously completed or published protocol, identify as such and list changes; otherwise, state plan for documenting important protocol amendments	n.a.
Support:			
Sources	5a	Indicate sources of financial or other support for the review	11
Sponsor	5b	Provide name for the review funder and/or sponsor	n.a.
Role of sponsor or funder	5c	Describe roles of funder(s), sponsor(s), and/or institution(s), if any, in developing the protocol	n.a.
**INTRODUCTION**			
Rationale	6	Describe the rationale for the review in the context of what is already known	3,4
Objectives	7	Provide an explicit statement of the question(s) the review will address with reference to participants, interventions, comparators, and outcomes (PICO)	4
**METHODS**			
Eligibility criteria	8	Specify the study characteristics (such as PICO, study design, setting, time frame) and report characteristics (such as years considered, language, publication status) to be used as criteria for eligibility for the review	5,6
Information sources	9	Describe all intended information sources (such as electronic databases, contact with study authors, trial registers or other grey literature sources) with planned dates of coverage	6
Search strategy	10	Present draft of search strategy to be used for at least one electronic database, including planned limits, such that it could be repeated	6, Table 1
Study records:			

For peer review only

Data management	11a	Describe the mechanism(s) that will be used to manage records and data throughout the review	7
Selection process	11b	State the process that will be used for selecting studies (such as two independent reviewers) through each phase of the review (that is, screening, eligibility and inclusion in meta-analysis)	7
Data collection process	11c	Describe planned method of extracting data from reports (such as piloting forms, done independently, in duplicate), any processes for obtaining and confirming data from investigators	7
Data items	12	List and define all variables for which data will be sought (such as PICO items, funding sources), any pre-planned data assumptions and simplifications	7
Outcomes and prioritization	13	List and define all outcomes for which data will be sought, including prioritization of main and additional outcomes, with rationale	6
Risk of bias in individual studies	14	Describe anticipated methods for assessing risk of bias of individual studies, including whether this will be done at the outcome or study level, or both; state how this information will be used in data synthesis	7
Data synthesis	15a	Describe criteria under which study data will be quantitatively synthesised	8
	15b	If data are appropriate for quantitative synthesis, describe planned summary measures, methods of handling data and methods of combining data from studies, including any planned exploration of consistency (such as I^2, Kendall's τ)	8
	15c	Describe any proposed additional analyses (such as sensitivity or subgroup analyses, meta-regression)	8
	15d	If quantitative synthesis is not appropriate, describe the type of summary planned	8
Meta-bias(es)	16	Specify any planned assessment of meta-bias(es) (such as publication bias across studies, selective reporting within studies)	8
Confidence in cumulative evidence	17	Describe how the strength of the body of evidence will be assessed (such as GRADE)	11

*** It is strongly recommended that this checklist be read in conjunction with the PRISMA-P Explanation and Elaboration (cite when available) for important clarification on the items. Amendments to a review protocol should be tracked and dated. The copyright for PRISMA-P (including checklist) is held by the PRISMA-P Group and is distributed under a Creative Commons Attribution Licence 4.0.**

From: Shamseer L, Moher D, Clarke M, Ghersi D, Liberati A, Petticrew M, Shekelle P, Stewart L, PRISMA-P Group. Preferred reporting items for systematic review and meta-analysis protocols (PRISMA-P) 2015: elaboration and explanation. BMJ. 2015 Jan 2;349(jan02 1):g7647.

For peer review only

BMJ Open

Effects of comparing 2 liters polyethylene glycol alone or plus ascorbic acid and 4 liters polyethylene glycol alone with each other for bowel preparation before colonoscopy: protocol for a systematic review and network meta-analysis

Journal:	*BMJ Open*
Manuscript ID	bmjopen-2017-018217.R1
Article Type:	Protocol
Date Submitted by the Author:	30-Aug-2017
Complete List of Authors:	Tian, Xu; Chongqing Cancer Hospital, Department of Gastroenterology<br>Chen, Wei-Qing; Chongqing Cancer Hospital, Department of Gastroenterology<br>Huang, Jie-Li; Chongqing Cancer Hospital, Department of Gastroenterology<br>He, Lan-Ying; Chongqing Cancer Hospital, Department of Gastroenterology<br>Liu, Bang-Lun; Chongqing Cancer Hospital, Department of Gastroenterology<br>Liu, Xi; Chongqing Cancer Hospital, Department of Gastroenterology<br>Zhou, Hang; Chongqing Cancer Hospital, Department of Gastroenterology<br>Liu, Bing-Rong; Chongqing Cancer Hospital, Department of Gastroenterology
**Primary Subject Heading**:	Evidence based practice
Secondary Subject Heading:	Gastroenterology and hepatology, Evidence based practice, Nursing, Oncology
Keywords:	Colonoscopy, Bowel preparation, Polyethylene glycol, Ascorbic acid, Meta-analysis

SCHOLARONE™
Manuscripts

For peer review only - http://bmjopen.bmj.com/site/about/guidelines.xhtml

Effects of comparing 2 liters polyethylene glycol alone or plus ascorbic acid and 4 liters polyethylene glycol alone with each other for bowel preparation before colonoscopy: protocol for a systematic review and network meta-analysis

Xu Tian[1*], Wei-Qing Chen[1*], Jie-Li Huang[1], Lan-Ying He[1], Bang-Lun Liu[1], Xi Liu[1], Hang Zhou[1], Bing-Rong Liu[2]

*These authors have contributed equally to this work as first author.

[1]Department of Gastroenterology, Chongqing Cancer Institute & Hospital & Cancer Center, Chongqing 400030, China

[2]Hospital of Digestive Diseases, the First Affiliated Hospital of Zhengzhou University, Zhengzhou 450052, China

Corresponding author: Wei-Qing Chen, MD, Department of Gastroenterology, Chongqing Cancer Institute & Hospital & Cancer Center, 181 Hanyu Road, Shapingba District, Chongqing 400030, China; telephone number: (86) 0 18222275023, e-mail: chenwq20140809@163.com

For peer review only

ABSTRACT

Introduction Colonoscopy has been regarded as a standard method of early detecting and removing gastrointestinal lesions, while adequate bowel preparation is the prerequisite of determining the diagnostic accuracy and treatment safety of this process. Polyethylene glycol (PEG)-based bowel preparation regime remains the first recommendation, but optimal option is still uncertainty. The aim of this systematic review and network meta-analysis of randomized controlled trials (RCTs) is to determine the optimal PEG-based bowel preparation regime before colonoscopy.

Methods and analysis We will assign two investigators to independently search all potential citations, screen records, abstract essential information, and appraise risk of bias accordingly. And then, random effects pairwise and network meta-analyses of RCTs comparing 2 liters PEG alone or plus ascorbic acid (Asc) and 4 liters PEG alone with each other will be performed by using RevMan 5.3 (Copenhagen: The Nordic Cochrane Centre, The Cochrane Collaboration, 2013), Stata 14 (StataCorp, Texas) and WinBUGS 1.4 (imperial College School of Medicine at St Mary's, London) from January 2000 to April 2017. The surface under the cumulative ranking curve (SCURA) will also be calculated in order to rank regimes.

Ethics and dissemination The ethics approval and patient written informed consent will not be required because of all analyses in the present study will be performed based on data from published studies. We will submit our systematic review and network meta-analysis to a peer-reviewed scientific journal for publication.

Systematic review registration PROSPRO: CRD42017068957

For peer review only - http://bmjopen.bmj.com/site/about/guidelines.xhtml

Strengths and limitations of this study

The protocol addresses the important question of which 2 liters PEG alone or plus Asc and 4 liters PEG alone regime offers the most benefits for bowel preparation efficacy before colonoscopy.

The present network meta-analysis has a clearly established aim, stringent inclusion criteria, state-of-the-art methods for data collection and quantitative synthesis.

The present network meta-analysis designed series of established methods to reduce the impact of heterogeneity and risk of bias on the pooled results.

The present network meta-analysis will rank all investigated PEG-based bowel preparation regimes in terms of each outcome, which facilitates evidence-informed decision-making.

Limitations include variations in administration times of drinking the same bowel preparation regimes, diet description prior to colonoscopy, type of colonoscopies, and assessment tool for bowel preparation efficacy.

For peer review only

BACKGROUND

Colorectal cancer (CRC) is one of the most common cancers diagnosed in the world and it is also the major contributor to cancer-associated morbidity and mortality [1]. Colonoscopy has been considered as the most effective method for early detection and prevention of CRC [2]. Published evidences suggested that early detection and endoscopic resection of polyps and abnormal lesions gastrointestinal tract can reduce approximately 50% mortality of CRC [3,4]. It is noted that, however, the adequate bowel preparation is the prerequisite of guaranteeing diagnostic accuracy and therapeutic safety of colonoscopy [5]. Issued data illustrated a sever fact that more than 40% of colonoscopy failures resulted from inadequate bowel preparation [6]. Moreover, inadequate bowel preparation also caused other negative consequences such as missed detection of polyps or lesions, increased risk of procedure-related complications, and increased economic costs [7]. Several factors can affect the quality of bowel preparation [8], and low patient-based compliance, poor palatability of bowel preparation solution, and inevitable requirement of drinking a large volume of preparation solution account for 20% to 25% inadequate bowel preparations [7]. However, low patient-based compliance with recommend regime play a decisive role in the overall success of the procedure [9].

For the purpose of improving quality of bowel preparation, several regimes have been developed such as polyethylene glycol (PEG)-based solutions, sodium phosphate (NaP), and sodium picosulfate solutions. Of these all regimes, PEG-based regimes are still first recommendation [10]. Several modified regimes, including split-dose regime,

For peer review only - http://bmjopen.bmj.com/site/about/guidelines.xhtml

low-volume regime, low-volume plus ascorbic acid (Asc), have been designed because patients are difficult to intake traditional 4L PEG owing to the large volume of fluid and poor palatability [11]. A series of randomized controlled trials (RCTs) have been performed to investigate the comparative efficacy of split dose versus single dose [12], low volume (2L) plus Asc versus traditional volume (4L) [13], and low volume plus Asc versus low volume [14]. However, the study regarding low volume versus traditional volume, low volume versus low volume plus Asc with split dose, and low volume versus traditional volume with split dose have not yet been identified. Moreover, individual study is difficult to identify subtle clinical differences owing to the smaller patient number [15]. Several meta-analyses have also been performed to evaluate the efficacy of low volume versus traditional volume [16], low volume versus plus Asc versus traditional volume [17], and split dose versus single dose [18,19]. Traditional meta-analysis methods, however, is unable to investigate the comparative efficacy of more than 2 interventions.

In order to solve the limitations of traditional meta-analysis technique, Bayesian network meta-analysis based on Markov Chain Monte Carlo (MCMC) and Gibbs Sampling, the expansion of pairwise meta-analysis, has been developed to evaluate the comparative efficacy of multiple treatments which are not directly compared in individual RCT [20]. And thus, we proposed this network meta-analysis to establish the effects of comparing 2 liters PEG alone or plus Asc and 4 liters PEG alone with each other prior to colonoscopy. We designed this systematic review and network meta-analysis on May 10, 2017; and we expected to complete this study on December

For peer review only - http://bmjopen.bmj.com/site/about/guidelines.xhtml

For peer review only

31, 2017.

METHODS AND DESIGN

We designed and completed this protocol for systematic review and network meta-analysis according to the preferred reporting items for systematic review and meta-analysis protocols (PRISMA-P) 2015: elaboration and explanation [21]. The systematic review and network meta-analysis was registered in International Prospective Register of Systematic Reviews (PROSPERO) (CRD42017068957). We will perform this traditional pairwise and network meta-analysis in accordance with the Cochrane Handbook for Systematic Reviews of Interventions [22] and report all results according to the preferred reporting items for systematic review and meta-analysis for network meta-analysis (PRISMA-NMA) [23].

Selection criteria

In our meta-analysis, the study will be considered if the following inclusion criteria are met: (i) Patients: all adult patients undergoing elective colonoscopy, irrespective of outpatients and inpatients; (ii) Intervention: all PEG-based bowel preparation regimes including 4L PEG and 2L PEG plus Asc with single or split dose and did not combine with other drugs, we drawn the possible evidence network according to the targeted regimes in terms of bowel preparation efficacy (see **Figure 1**); (iii) Outcomes: bowel preparation efficacy (BPE) was regarded as primary outcome, and the secondary outcomes including compliance with recommend regime (CP), preference to repeat

For peer review only - http://bmjopen.bmj.com/site/about/guidelines.xhtml

the same regime (PRSR), acceptance to regime (AT), adverse events (AEs), and detection rate of polyps and adenomas (DRPA); (iv) Study design: only RCTs were included, abstract with sufficient data was also considered; and (vi) Language: only full-text published in English Language will be included.

Study will be excluded if it met at least one of following criteria: (i) essential information cannot be extracted; (ii) duplication with poor methodology and insufficient data; (iii) non-original research types such as review, editorial, letter to the editor and comments; and (iv) the study investigating the potential of bowel preparation regime in special patients such as elderly or previous poor bowel preparation.

Definition of outcomes

In our systemic review and network meta-analysis, the BPE was also regarded as successful bowel preparation, and it was defined an Ottawa score of < 5 or a Boston Bowel Preparation Scale (BBPS) score of $\geqslant$ 2 for all locations or an excellent or good bowel preparation designation on the Aronchik scale or other non-validated 3-, 4-, or 5-point scales (excellent, good, fair, poor, very poor). CP was defined as adherence to the bowel preparation prescribed or consumption of at least 75% of the prescribed bowel preparation. PRSR, AT and AEs were measured by using the specified questionnaires in each eligible study (i.e. defined by individual study). DRPA refers to the number of detecting actually polyps and adenomas.

For peer review only - http://bmjopen.bmj.com/site/about/guidelines.xhtml

Identification of citations

We will firstly electronically search the PubMed, Cochrane Central Register of Controlled Trials (CENTRAL), and EMBASE in order to capture all potential records investigating the comparative efficacy of different PEG-based bowel preparation regimes from January 2000 to April 2017. "*Colonoscopy*", "*polyethylene glycols*", and "*random*" were used to construct search algorithms in accordance with the requests of targeted databases, and all possible search algorithms have been documented in electronical supplementary material Table 1 **(ESM-Table 1)**.

After electronic search, we will also hand check the reference lists of all eligible studies and topic-related review and electronically retrieve the Clinicaltrial.gov for the purpose of covering all potential eligible study. It is noted that, however, only studies published in English will be considered in our systematic review and network meta-analysis.

Data extraction

We have designed a standard data extraction form before performed our previous two systematic reviews and network meta-analyses (see electronical supplementary material-SDE). All captured citations will be imposed into EndNote literature management software version X7. We will consequently assign two reviewers to abstract the basic information and data for specific outcome from eligible study, such as first author, publication year, age of participants, sample size, bowel preparation regimes, and outcomes of interest using this standard data extraction form [24]. We will

For peer review only - http://bmjopen.bmj.com/site/about/guidelines.xhtml

contact the corresponding author if sufficient data of an eligible study cannot be abstracted from full-text. The Kappa value will be calculated in order to assess the inter-investigator reliability. We will establish the consensus principle to be the method of resolving divergences between reviewers.

Quality assessment of individual study

We will assign two independent reviewers to appraise the risk of bias from seven domains including randomization sequence generation, allocation concealment, blinding of participants, blinding of study personnel, blinding of outcome assessors, incomplete outcome data, selective reporting and other bias with the Cochrane risk of bias assessment tool [22,25]. A study will be assigned into a level of '*high risk of bias*', '*unclear risk of bias*' or '*low risk of bias*' according to the match level between actual information and evaluation criteria [22].

Description of the available data

We derived each pairwise comparison from descriptive statistics on available data and selected variables for study and population characteristics such as age, study length and outcome-relevant baseline risk factors. A network diagram was used for each outcome to present the direct comparisons between different bowel preparation regimes and control groups. In these diagrams, nodes (circles) represented various bowel preparations and their sizes were proportional to the sample size of each respective intervention; edges (lines) indicated direct comparisons and their

For peer review only - http://bmjopen.bmj.com/site/about/guidelines.xhtml

thicknesses were proportional to the standard error (precision).

Statistical analysis

We will firstly perform traditional pairwise meta-analysis based on random effect model, which incorporates within- and between-studies heterogeneity, to estimate the summarized odd ratio (OR) and 95% confidence intervals (CIs) [26]. Chi^2 method will be adopted to test the heterogeneity [27] and I^2 statistic will be used to estimate the proportion of the overall variation that is attributable to between-study heterogeneity [28]. The value of I^2 statistic is larger than 50% indicating substantial heterogeneity [28]. We will draw the funnel plot to identify publication bias if the number of studies analyzed was more than 10 [29]. The studies with more than two comparison groups will be quantitatively incorporated in pairwise meta-analysis according to the specific comparison.

Following the traditional pairwise meta-analysis, random-effects network meta-analysis will be performed according to the methods described by Chaimani and colleagues [30]. The initial values, automatically generated from software, will be used to fit the model [31]. We plan to perform 70000 iterations and 30000 burn-in for each outcome and convergence.

The surface under the cumulative ranking curve (SUCRA) will also be drawn in order to rank all PEG-based bowel preparation regimes and a higher value suggests better results for respective regime [32].

All analyses will be conducted by using the RevMan 5.3 (Copenhagen: The Nordic

For peer review only - http://bmjopen.bmj.com/site/about/guidelines.xhtml

Cochrane Centre, The Cochrane Collaboration, 2013), Stata 14 (StataCorp, Texas) and WinBUGS 1.4 (imperial College School of Medicine at St Mary's, London).

Assessment of small study effects and inconsistency

We will generate the comparison-adjusted funnel plot to assess the small-study effects when the number of studies included in one pair of comparison was more than 10 [33]. We will calculate the inconsistency factor based on the loop-specific method to assess the inconsistency [30].

Subgroup and sensitivity analyses

In case of possible important heterogeneity or inconsistency, we explored the possible sources using subgroup and meta-regression analyses. Subgroup analyses were planned for time of colonoscopy, patient sources and age. Sensitivity analyses were planned for bowel preparation quality by analyzing only studies considered being at low risk of bias.

DISCUSSION

The CRC is one of the most common malignancies, and issued statistics illustrated that it is the fourth contributor to the cancer-death worldwide [1]. Colonoscopy has been regarded as the standard process of early preventing and detecting CRC in clinical practice [2]. However, diagnostic accuracy and operation safety during performing colonoscopy mainly depend on the quality of bowel preparation [34]. Although several

For peer review only

novel bowel preparation regimes have been developed in order to improve the tolerability and compliance of patients, the PEG-based regimes have been first-line recommendation [10]. It is noted that several modified regimes have been applied in clinical practice, but no primary study and traditional pairwise meta-analysis comparing various PEG-based bowel preparation regimes with each other has been published. And thus it is still debate which PEG-based regime should be optimally described. We hence proposed this network meta-analysis to determine the optimal PEG-based regime for the purpose of facilitating the informed-decision making.

This network meta-analysis was one of the first to compare the direct and indirect effects of different PEG-based regimes for bowel preparation prior to colonoscopy. The results of the present network meta-analysis will influence evidence-based decision-making in bowel preparation regime prescription, since it will be fundamental for reliable recommendations in the consideration of bowel preparation regime before colonoscopy.

ACKNOWLEDGMENTS

We would like to appreciate the editor and anonymous referees for their helps.

AUTHORS CONTRIBUTIONS

X.T. and W.-Q.C. conceived and designed this study; X.T. and J.-L.H. searched and selected studies; L.-Y.H. and B.-L.L. extracted essential information; X.T. and B.-R.L. assessed the risk of bias; X.T., W.-Q.C., X.L. and H.Z. performed statistical analyses;

For peer review only

X.T. and W.-Q.C. interpreted the pooled results; X.T., W.-Q.C. and B.-R.L. drafted the manuscript; all authors approved this manuscript to be considered for publication.

FUNDING STATEMENT

This research received no specific grant from any funding agency in the public, commercial or not-for-profit sectors.

COMPETING INTERESTS STATEMENT

The authors report no declarations of interest.

For peer review only

For peer review only - http://bmjopen.bmj.com/site/about/guidelines.xhtml

REFERENCES

1. Torre LA, Bray F, Siegel RL, Ferlay J, Lortet-Tieulent J, Jemal A. Global cancer statistics, 2012. *CA Cancer J Clin.* Mar 2015;65(2):87-108.
2. Lieberman DA, Weiss DG, Bond JH, Ahnen DJ, Garewal H, Chejfec G. Use of colonoscopy to screen asymptomatic adults for colorectal cancer. Veterans Affairs Cooperative Study Group 380. *The New England journal of medicine.* Jul 20 2000;343(3):162-168.
3. Quintero E, Castells A, Bujanda L, et al. Colonoscopy versus fecal immunochemical testing in colorectal-cancer screening. *The New England journal of medicine.* Feb 23 2012;366(8):697-706.
4. Zauber AG, Winawer SJ, O'Brien MJ, et al. Colonoscopic polypectomy and long-term prevention of colorectal-cancer deaths. *The New England journal of medicine.* Feb 23 2012;366(8):687-696.
5. Marmo R, Rotondano G, Riccio G, et al. Effective bowel cleansing before colonoscopy: a randomized study of split-dosage versus non-split dosage regimens of high-volume versus low-volume polyethylene glycol solutions. *Gastrointestinal endoscopy.* Aug 2010;72(2):313-320.
6. Ponchon T, Boustière C, Heresbach D, Hagege H, Tarrerias A, Halphen M. A low-volume polyethylene glycol plus ascorbate solution for bowel cleansing prior to colonoscopy: the NORMO randomised clinical trial. *Digestive and liver disease : official journal of the Italian Society of Gastroenterology and the Italian Association for the Study of the Liver.* 2013;45(10):820-826. http://onlinelibrary.wiley.com/o/cochrane/clcentral/articles/982/CN-01122982/frame.html.
7. Froehlich F, Wietlisbach V, Gonvers JJ, Burnand B, Vader JP. Impact of colonic cleansing on quality and diagnostic yield of colonoscopy: the European Panel of Appropriateness of Gastrointestinal Endoscopy European multicenter study. *Gastrointestinal endoscopy.* Mar 2005;61(3):378-384.
8. Kaminski MF, Regula J, Kraszewska E, et al. Quality indicators for colonoscopy and the risk of interval cancer. *The New England journal of medicine.* May 13 2010;362(19):1795-1803.
9. Ness RM, Manam R, Hoen H, Chalasani N. Predictors of inadequate bowel preparation for colonoscopy. *Am J Gastroenterol.* Jun 2001;96(6):1797-1802.
10. Mathus-Vliegen E, Pellise M, Heresbach D, et al. Consensus guidelines for the use of bowel preparation prior to colonic diagnostic procedures: colonoscopy and small bowel video capsule endoscopy. *Current medical research and opinion.* Aug 2013;29(8):931-945.
11. Ell C, Fischbach W, Bronisch HJ, et al. Randomized trial of low-volume PEG solution versus standard PEG + electrolytes for bowel cleansing before colonoscopy. *Am J Gastroenterol.* Apr 2008;103(4):883-893.
12. Kim M, Park J, Park J, et al. Does Polyethylene Glycol (PEG) Plus Ascorbic Acid Induce More Mucosal Injuries than Split-Dose 4-L PEG during Bowel Preparation? *Gut and liver.* 2017;10(2):237-243. http://onlinelibrary.wiley.com/o/cochrane/clcentral/articles/765/CN-01260765/frame.html.
13. Rivas J, Perez A, Hernandez M, Schneider A, Castro F. Efficacy of morning-only 4 liter sulfa free polyethylene glycol vs 2 liter polyethylene glycol with ascorbic acid for afternoon colonoscopy. *World journal of gastroenterology.* 2014;20(30):10620-10627.

For peer review only - http://bmjopen.bmj.com/site/about/guidelines.xhtml

http://onlinelibrary.wiley.com/o/cochrane/clcentral/articles/049/CN-01040049/frame.html.
14. Kanie H, Kojima I, Araki H, et al. A randomized clinical trial of low-volume polyethylene glycol versus low-volume polyethylene glycol with ascorbic acid solutions. *Gastrointestinal endoscopy.* 2016;83(5):AB226.
15. Wang J, Meng XH, Guo ZM, Wu YH, Zhao JG. Interventions for treating displaced midshaft clavicular fractures: a Bayesian network meta-analysis of randomized controlled trials. *Medicine (Baltimore).* Mar 2015;94(11):e595.
16. Godfrey J, Clark R, Choudhary A, Ashraf I, Matteson M, Bechtold M. Low-volume polyethylene glycol and ascorbic acid for bowel preparation prior to colonoscopy: A meta-analysis. *American Journal of Gastroenterology.* 2011;106:S527.
17. Xie Q, Chen L, Zhao F, et al. A meta-analysis of randomized controlled trials of low-volume polyethylene glycol plus ascorbic acid versus standard-volume polyethylene glycol solution as bowel preparations for colonoscopy. *PloS one.* 2014;9(6):e99092.
18. Avalos DJ, Sarkis FS, Keihanian T, et al. Same-day morning bowel regimens vs. Split regimens for colonoscopy: A meta-analysis of randomized controlled trials. *Gastroenterology.* 2016;150(4):S839.
19. Ménard C, Barkun AN, Martel M, Restellini S, Kherad O. Split-dose versus same-day bowel preparations for colonoscopy: A meta-analysis. *Gastroenterology.* 2016;150(4):S838.
20. Mills EJ, Ioannidis JP, Thorlund K, Schunemann HJ, Puhan MA, Guyatt GH. How to use an article reporting a multiple treatment comparison meta-analysis. *Jama.* Sep 26 2012;308(12):1246-1253.
21. Shamseer L, Moher D, Clarke M, et al. Preferred reporting items for systematic review and meta-analysis protocols (PRISMA-P) 2015: elaboration and explanation. *BMJ.* Jan 02 2015;349:g7647.
22. Higgins JP, Green S. Cochrane Handbook For Systematic Reviews Of Interventions Version 5.0.0. *Naunyn-Schmiedebergs Archiv für experimentelle Pathologie und Pharmakologie.* 2009;210(2):S38.
23. Hutton B, Salanti G, Caldwell DM, et al. The PRISMA extension statement for reporting of systematic reviews incorporating network meta-analyses of health care interventions: checklist and explanations. *Ann Intern Med.* Jun 02 2015;162(11):777-784.
24. Song GM, Tian X, Zhang L, et al. Immunonutrition Support for Patients Undergoing Surgery for Gastrointestinal Malignancy: Preoperative, Postoperative, or Perioperative? A Bayesian Network Meta-Analysis of Randomized Controlled Trials. *Medicine (Baltimore).* Jul 2015;94(29):e1225.
25. Zeng X, Zhang Y, Kwong JS, et al. The methodological quality assessment tools for preclinical and clinical studies, systematic review and meta-analysis, and clinical practice guideline: a systematic review. *J Evid Based Med.* Feb 2015;8(1):2-10.
26. DerSimonian R, Laird N. Meta-analysis in clinical trials. *Controlled clinical trials.* Sep 1986;7(3):177-188.
27. Bowden J, Tierney JF, Copas AJ, Burdett S. Quantifying, displaying and accounting for heterogeneity in the meta-analysis of RCTs using standard and generalised Q statistics. *BMC medical research methodology.* 2011;11:41.
28. Higgins JPT, Thompson SG. Quantifying heterogeneity in a meta-analysis. *Statistics in Medicine.* 2002;21(11):1539-1558.

For peer review only

For peer review only - http://bmjopen.bmj.com/site/about/guidelines.xhtml

29. Tian X, Zhou JG, Zeng Z, et al. Cetuximab in patients with esophageal cancer: a systematic review and meta-analysis of randomized controlled trials. *Medical Oncology.* 2015;32(4):1-10.

30. Chaimani A, Higgins JPT, Mavridis D, Spyridonos P, Salanti G. Graphical Tools for Network Meta-Analysis in STATA. *Plos One.* 2013;8(10):e76654-e76654.

31. Sutton DA, Ades AE, Cooper N, Abrams K. Use of Indirect and Mixed Treatment Comparisons for Technology Assessment. *Pharmacoeconomics.* 2008;26(9):753-767.

32. Georgia S, Ades AE, Ioannidis JPA. Graphical methods and numerical summaries for presenting results from multiple-treatment meta-analysis: an overview and tutorial. *Journal of Clinical Epidemiology.* 2011;64(2):163-171.

33. Chen LX, Zhou ZR, Li YL, et al. Comparison of Bone Mineral Density in Lumbar Spine and Fracture Rate among Eight Drugs in Treatments of Osteoporosis in Men: A Network Meta-Analysis. *Plos One.* 2015;10(5).

34. Rapier R, Houston C. A prospective study to assess the efficacy and patient tolerance of three bowel preparations for colonoscopy. *Gastroenterology nursing : the official journal of the Society of Gastroenterology Nurses and Associates.* Jul-Aug 2006;29(4):305-308.

For peer review only

For peer review only - http://bmjopen.bmj.com/site/about/guidelines.xhtml

FIGURE LENGENDS

Figure 1 Possible evidence network of all possible PEG-based bowel preparation regimes in terms of bowel preparation efficacy. The yellow solid line indicates direct comparisons between regimes which were directly compared in original studies. The brown node represents the each PEG-based bowel preparation regime. PEG = polyethylene glycol, SD = split dose, Asc = ascorbic acid.

For peer review only

For peer review only - http://bmjopen.bmj.com/site/about/guidelines.xhtml

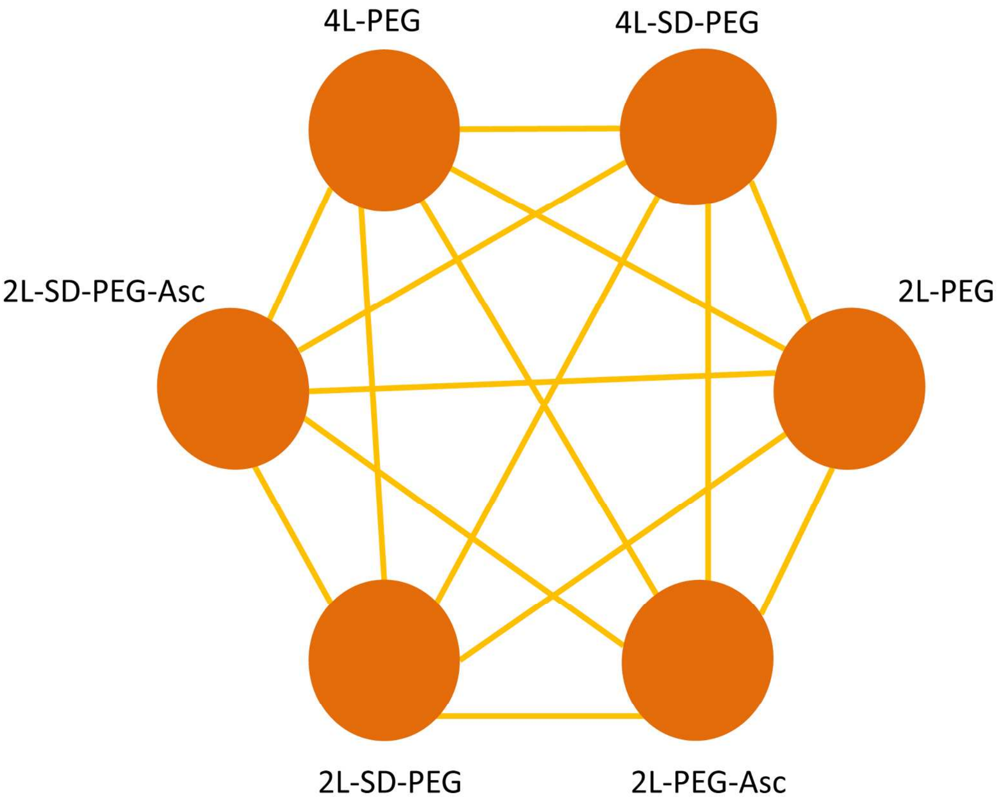

The yellow solid line indicates direct comparisons between regimes which were directly compared in original studies. The brown node represents the each PEG-based bowel preparation regime. PEG = polyethylene glycol, SD = split dose, Asc = ascorbic acid.

185x148mm (300 x 300 DPI)

CENTRAL Search Algorithm

ID	Search
#1	Polyethylene Glycol*:ti,ab,kw or Macrogol*:ti,ab,kw or Glycol, Polyethylene:ti,ab,kw or Glycols, Polyethylene:ti,ab,kw or Polyethylene Oxide*:ti,ab,kw (Word variations have been searched)
#2	Oxide, Polyethylene:ti,ab,kw or Oxides, Polyethylene:ti,ab,kw or Polyethyleneoxide*:ti,ab,kw or Polyoxyethylene*:ti,ab,kw or Tritons:ti,ab,kw (Word variations have been searched)
#3	MeSH descriptor: [Polyethylene Glycols] explode all trees
#4	#1 or #2 or #3
#5	Colonoscop*:ti,ab,kw or Colonoscopic Surgical Procedure*:ti,ab,kw or Procedure, Colonoscopic Surgical:ti,ab,kw or Procedures, Colonoscopic Surgical:ti,ab,kw or Surgical Procedure, Colonoscopic:ti,ab,kw (Word variations have been searched)
#6	Surgery, Colonoscopic:ti,ab,kw or Surgical Procedures, Colonoscopic:ti,ab,kw or Colonoscopic Surger*:ti,ab,kw or Surgeries, Colonoscopic:ti,ab,kw (Word variations have been searched)
#7	MeSH descriptor: [Colonoscopy] explode all trees
#8	#5 or #6 or #7
#9	random*:ti,ab,kw (Word variations have been searched)
#10	MeSH descriptor: [Randomized Controlled Trial] explode all trees
#11	MeSH descriptor: [Randomized Controlled Trials as Topic] explode all trees
#12	#9 or #10 or #11
#13	#4 and #8 and #12

For peer review only

PubMed Search Algorithm

Search	Query
#8	Search (((("Polyethylene Glycols"[Mesh]) OR (((((((((Polyethylene Glycol*[Title/Abstract]) OR Macrogol*[Title/Abstract]) OR Glycol, Polyethylene[Title/Abstract]) OR Glycols, Polyethylene[Title/Abstract]) OR Polyethylene Oxide*[Title/Abstract]) OR Oxide, Polyethylene[Title/Abstract]) OR Oxides, Polyethylene[Title/Abstract]) OR Polyethyleneoxide*[Title/Abstract]) OR Polyoxyethylene*[Title/Abstract]) OR Tritons[Title/Abstract]))) AND (("Colonoscopy"[Mesh]) OR (((((((((Colonoscop*[Title/Abstract]) OR Colonoscopic Surgical Procedure*[Title/Abstract]) OR Procedure, Colonoscopic Surgical[Title/Abstract]) OR Procedures, Colonoscopic Surgical[Title/Abstract]) OR Surgical Procedure, Colonoscopic[Title/Abstract]) OR Surgery, Colonoscopic[Title/Abstract]) OR Surgical Procedures, Colonoscopic[Title/Abstract]) OR Colonoscopic Surger*[Title/Abstract]) OR Surgeries, Colonoscopic[Title/Abstract]))) AND random*[Title/Abstract] Sort by: PublicationDate
#7	Search random*[Title/Abstract] Sort by: PublicationDate
#6	Search ("Colonoscopy"[Mesh]) OR (((((((((Colonoscop*[Title/Abstract]) OR Colonoscopic Surgical Procedure*[Title/Abstract]) OR Procedure, Colonoscopic Surgical[Title/Abstract]) OR Procedures, Colonoscopic Surgical[Title/Abstract]) OR Surgical Procedure, Colonoscopic[Title/Abstract]) OR Surgery, Colonoscopic[Title/Abstract]) OR Surgical Procedures, Colonoscopic[Title/Abstract]) OR Colonoscopic Surger*[Title/Abstract]) OR Surgeries, Colonoscopic[Title/Abstract]) Sort by: PublicationDate
#5	Search ((((((((Colonoscop*[Title/Abstract]) OR Colonoscopic Surgical Procedure*[Title/Abstract]) OR Procedure, Colonoscopic Surgical[Title/Abstract]) OR Procedures, Colonoscopic Surgical[Title/Abstract]) OR Surgical Procedure, Colonoscopic[Title/Abstract]) OR Surgery, Colonoscopic[Title/Abstract]) OR Surgical Procedures, Colonoscopic[Title/Abstract]) OR Colonoscopic Surger*[Title/Abstract]) OR Surgeries, Colonoscopic[Title/Abstract] Sort by: PublicationDate
#4	Search "Colonoscopy"[Mesh] Sort by: PublicationDate
#3	Search ("Polyethylene Glycols"[Mesh]) OR (((((((((Polyethylene Glycol*[Title/Abstract]) OR Macrogol*[Title/Abstract]) OR Glycol, Polyethylene[Title/Abstract]) OR Glycols, Polyethylene[Title/Abstract]) OR Polyethylene Oxide*[Title/Abstract]) OR Oxide, Polyethylene[Title/Abstract]) OR Oxides, Polyethylene[Title/Abstract]) OR Polyethyleneoxide*[Title/Abstract]) OR Polyoxyethylene*[Title/Abstract]) OR Tritons[Title/Abstract]) Sort by: PublicationDate
#2	Search (((((((((Polyethylene Glycol*[Title/Abstract]) OR Macrogol*[Title/Abstract]) OR Glycol, Polyethylene[Title/Abstract]) OR Glycols, Polyethylene[Title/Abstract]) OR Polyethylene Oxide*[Title/Abstract]) OR Oxide, Polyethylene[Title/Abstract]) OR Oxides, Polyethylene[Title/Abstract]) OR Polyethyleneoxide*[Title/Abstract]) OR Polyoxyethylene*[Title/Abstract]) OR Tritons[Title/Abstract] Sort by: PublicationDate
#1	Search "Polyethylene Glycols"[Mesh] Sort by: PublicationDate

For peer review only

Embase Search Algorithm

No. Query

#10. polyethylene:ab,ti AND glycol*:ab,ti OR macrogol*:ab,ti OR (glycol,:ab,ti AND polyethylene:ab,ti) OR (glycols,:ab,ti AND polyethylene:ab,ti) OR (polyethylene:ab,ti AND oxide*:ab,ti) OR (oxide,:ab,ti AND polyethylene:ab,ti) OR (oxides,:ab,ti AND polyethylene:ab,ti) OR polyethyleneoxide*:ab,ti OR polyoxyethylene:ab,ti OR tritons:ab,ti OR 'macrogol derivative'/exp AND (colonoscop*:ab,ti OR (colonoscopic:ab,ti AND surgical:ab,ti AND procedure*:ab,ti) OR (procedure,:ab,ti AND colonoscopic:ab,ti AND surgical:ab,ti) OR (procedures,:ab,ti AND colonoscopic:ab,ti AND surgical:ab,ti) OR (surgical:ab,ti AND procedure,:ab,ti AND colonoscopic:ab,ti) OR (surgery,:ab,ti AND colonoscopic:ab,ti) OR (surgical:ab,ti AND procedures,:ab,ti AND colonoscopic:ab,ti) OR (colonoscopic:ab,ti AND surger*:ab,ti) OR (surgeries,:ab,ti AND colonoscopic:ab,ti) OR 'colonoscopy'/exp) AND (random*:ab,ti OR 'randomized controlled trial'/exp OR 'randomized controlled trial (topic)'/exp)

#9. random*:ab,ti OR 'randomized controlled trial'/exp OR 'randomized controlled trial (topic)'/exp

#8. 'randomized controlled trial'/exp OR 'randomized controlled trial (topic)'/exp

#7. random*:ab,ti

#6. colonoscop*:ab,ti OR (colonoscopic:ab,ti AND surgical:ab,ti AND procedure*:ab,ti) OR (procedure,:ab,ti AND colonoscopic:ab,ti AND surgical:ab,ti) OR (procedures,:ab,ti AND colonoscopic:ab,ti AND surgical:ab,ti) OR (surgical:ab,ti AND procedure,:ab,ti AND colonoscopic:ab,ti) OR (surgery,:ab,ti AND colonoscopic:ab,ti) OR (surgical:ab,ti AND procedures,:ab,ti AND colonoscopic:ab,ti) OR (colonoscopic:ab,ti AND surger*:ab,ti) OR (surgeries,:ab,ti AND colonoscopic:ab,ti) OR 'colonoscopy'/exp

#5. 'colonoscopy'/exp

#4. colonoscop*:ab,ti OR (colonoscopic:ab,ti AND surgical:ab,ti AND procedure*:ab,ti) OR (procedure,:ab,ti AND colonoscopic:ab,ti AND surgical:ab,ti) OR (procedures,:ab,ti AND colonoscopic:ab,ti AND surgical:ab,ti) OR (surgical:ab,ti AND procedure,:ab,ti AND colonoscopic:ab,ti) OR (surgery,:ab,ti AND colonoscopic:ab,ti) OR (surgical:ab,ti AND

For peer review only

procedures,:ab,ti AND colonoscopic:ab,ti) OR (colonoscopic:ab,ti AND surger*:ab,ti) OR (surgeries,:ab,ti AND colonoscopic:ab,ti)

#3. polyethylene:ab,ti AND glycol*:ab,ti OR macrogol*:ab,ti OR (glycol,:ab,ti AND polyethylene:ab,ti) OR (glycols,:ab,ti AND polyethylene:ab,ti) OR (polyethylene:ab,ti AND oxide*:ab,ti) OR (oxide,:ab,ti AND polyethylene:ab,ti) OR (oxides,:ab,ti AND polyethylene:ab,ti) OR polyethyleneoxide*:ab,ti OR polyoxyethylene:ab,ti OR tritons:ab,ti OR 'macrogol derivative'/exp

#2. 'macrogol derivative'/exp

#1. polyethylene:ab,ti AND glycol*:ab,ti OR macrogol*:ab,ti OR (glycol,:ab,ti AND polyethylene:ab,ti) OR (glycols,:ab,ti AND polyethylene:ab,ti) OR (polyethylene:ab,ti AND oxide*:ab,ti) OR (oxide,:ab,ti AND polyethylene:ab,ti) OR (oxides,:ab,ti AND polyethylene:ab,ti) OR polyethyleneoxide*:ab,ti OR polyoxyethylene:ab,ti OR tritons:ab,ti

For peer review only

Basic Characteristics of Included into Study

Study Information	Author	Year	Country

Section	Item		
Methods	Allocation		
	Duration		
	Blinding		
	Location		
Participants	Diagnosis	Pts were diagnosed as GC based histology.	
	Age	Study Group	Control Group
	Sex	Study Group	Control Group
	Length of Illness	Study Group	Control Group
	Inclusion criteria		
	Exclusion criteria		
Interventions	Treatment Group – Content		
	Control group – Content		
Outcomes			
Notes			

Drop-outs	Drop out due to	Study group	Control group
	the numbers of patients in the early stage		
	the numbers of patients in the late stage		

Binary Data			
Outcomes	Name of Outcome	Event number	Total number
	Study group		
	Control group		

Binary Data			
Outcomes	Name of Outcome	Event number	Total number
	Study group		
	Control group		

Binary Data			
Outcomes	Name of Outcome	Event number	Total number
	Study group		
	Control group		

Continuous Data				
Outcomes	Name of outcome	Data extraction		
		Median	Range	P
	ECT group			
	Paroxetin group			

Continuous Data				
Outcomes	Name of outcome	Data extraction		
		Median	Range	P
	ECT group			
	Paroxetin group			

Assessing of Risk of Bias Tool		
**Item**	**Description**	**Risk of Bias**
**Sequence Generation**	Describe the method used to generate the allocation sequence in sufficient detail to allow an assessment of whether it should produce comparable groups	Was the allocation sequence adequately generated?
	Comment:	Low Risk
**Allocation Concealment**	Describe the method used to conceal the allocation sequence in sufficient detail to determine whether intervention allocations could have been foreseen	Was the allocation adequately concealed?
	Comment:	Low Risk
**Blinding of Participants and Personnel**	Describe all measures used, if any, to blind study participants and personnel from knowledge of which intervention a participant received. Provide any information relating to whether the intended blinding was effective	Was knowledge of the allocated intervention adequately prevented during the study?
	Comment:	Low Risk
**Blinding of Outcome Assessors**	Describe all measures used, if any, to blind outcome assessors from knowledge of which intervention a participant received. Provide any information relating to whether the intended blinding was effective.	Was knowledge of the allocated intervention adequately prevented during the study?
	Comment:	Unclear
**Incomplete Outcome Data**	Describe the completeness of outcome data for each main outcome, including attrition and exclusions from the analysis. State whether attrition and exclusions were reported, the numbers in each intervention group (compared with total randomized participants), reasons for attrition/exclusions where reported, and	Were incomplete outcome data adequately addressed?
	Comment:	Low Risk
**Selective Outcome Reporting**	State how the possibility of selective outcome reporting was examined by the review authors and what was found.	Are reports of the study free of suggestion of selective
	Comment:	Low Risk
**Other Bias**	State any important concerns about bias not addressed in the other domains in the tool. If particular questions/entries were re-specified in the review protocol, responses should be provided for each question/entry	Was the study apparently free of other problems that could put it at high risk of bias?
	Comment:	Low Risk

For peer review only

PRISMA-P (Preferred Reporting Items for Systematic review and Meta-Analysis Protocols) 2015 checklist: recommended items to address in a systematic review protocol*

Section and topic	Item No	Checklist item	Page
**ADMINISTRATIVE INFORMATION**			
Title:			
Identification	1a	Identify the report as a protocol of a systematic review	1
Update	1b	If the protocol is for an update of a previous systematic review, identify as such	n.a.
Registration	2	If registered, provide the name of the registry (such as PROSPERO) and registration number	n.a.
Authors:			
Contact	3a	Provide name, institutional affiliation, e-mail address of all protocol authors; provide physical mailing address of corresponding author	1
Contributions	3b	Describe contributions of protocol authors and identify the guarantor of the review	10,11
Amendments	4	If the protocol represents an amendment of a previously completed or published protocol, identify as such and list changes; otherwise, state plan for documenting important protocol amendments	n.a.
Support:			
Sources	5a	Indicate sources of financial or other support for the review	11
Sponsor	5b	Provide name for the review funder and/or sponsor	n.a.
Role of sponsor or funder	5c	Describe roles of funder(s), sponsor(s), and/or institution(s), if any, in developing the protocol	n.a.
**INTRODUCTION**			
Rationale	6	Describe the rationale for the review in the context of what is already known	3,4
Objectives	7	Provide an explicit statement of the question(s) the review will address with reference to participants, interventions, comparators, and outcomes (PICO)	4
**METHODS**			
Eligibility criteria	8	Specify the study characteristics (such as PICO, study design, setting, time frame) and report characteristics (such as years considered, language, publication status) to be used as criteria for eligibility for the review	5,6
Information sources	9	Describe all intended information sources (such as electronic databases, contact with study authors, trial registers or other grey literature sources) with planned dates of coverage	6
Search strategy	10	Present draft of search strategy to be used for at least one electronic database, including planned limits, such that it could be repeated	6, Table 1
Study records:			

For peer review only

Data management	11a	Describe the mechanism(s) that will be used to manage records and data throughout the review	7
Selection process	11b	State the process that will be used for selecting studies (such as two independent reviewers) through each phase of the review (that is, screening, eligibility and inclusion in meta-analysis)	7
Data collection process	11c	Describe planned method of extracting data from reports (such as piloting forms, done independently, in duplicate), any processes for obtaining and confirming data from investigators	7
Data items	12	List and define all variables for which data will be sought (such as PICO items, funding sources), any pre-planned data assumptions and simplifications	7
Outcomes and prioritization	13	List and define all outcomes for which data will be sought, including prioritization of main and additional outcomes, with rationale	6
Risk of bias in individual studies	14	Describe anticipated methods for assessing risk of bias of individual studies, including whether this will be done at the outcome or study level, or both; state how this information will be used in data synthesis	7
Data synthesis	15a	Describe criteria under which study data will be quantitatively synthesised	8
	15b	If data are appropriate for quantitative synthesis, describe planned summary measures, methods of handling data and methods of combining data from studies, including any planned exploration of consistency (such as I^2, Kendall's τ)	8
	15c	Describe any proposed additional analyses (such as sensitivity or subgroup analyses, meta-regression)	8
	15d	If quantitative synthesis is not appropriate, describe the type of summary planned	8
Meta-bias(es)	16	Specify any planned assessment of meta-bias(es) (such as publication bias across studies, selective reporting within studies)	8
Confidence in cumulative evidence	17	Describe how the strength of the body of evidence will be assessed (such as GRADE)	11

*** It is strongly recommended that this checklist be read in conjunction with the PRISMA-P Explanation and Elaboration (cite when available) for important clarification on the items. Amendments to a review protocol should be tracked and dated. The copyright for PRISMA-P (including checklist) is held by the PRISMA-P Group and is distributed under a Creative Commons Attribution Licence 4.0.**

From: Shamseer L, Moher D, Clarke M, Ghersi D, Liberati A, Petticrew M, Shekelle P, Stewart L, PRISMA-P Group. Preferred reporting items for systematic review and meta-analysis protocols (PRISMA-P) 2015: elaboration and explanation. BMJ. 2015 Jan 2;349(jan02 1):g7647.

For peer review only

BMJ Open

Effects of comparing 2 liters polyethylene glycol alone or plus ascorbic acid and 4 liters polyethylene glycol alone with each other for bowel preparation before colonoscopy: protocol for a systematic review and network meta-analysis

Journal:	*BMJ Open*
Manuscript ID	bmjopen-2017-018217.R2
Article Type:	Protocol
Date Submitted by the Author:	18-Sep-2017
Complete List of Authors:	Tian, Xu; Chongqing Cancer Hospital, Department of Gastroenterology<br>Chen, Wei-Qing; Chongqing Cancer Hospital, Department of Gastroenterology<br>Huang, Jie-Li; Chongqing Cancer Hospital, Department of Gastroenterology<br>He, Lan-Ying; Chongqing Cancer Hospital, Department of Gastroenterology<br>Liu, Bang-Lun; Chongqing Cancer Hospital, Department of Gastroenterology<br>Liu, Xi; Chongqing Cancer Hospital, Department of Gastroenterology<br>Zhou, Hang; Chongqing Cancer Hospital, Department of Gastroenterology<br>Liu, Bing-Rong; Chongqing Cancer Hospital, Department of Gastroenterology
**Primary Subject Heading**:	Evidence based practice
Secondary Subject Heading:	Gastroenterology and hepatology, Evidence based practice, Nursing, Oncology
Keywords:	Colonoscopy, Bowel preparation, Polyethylene glycol, Ascorbic acid, Meta-analysis

SCHOLARONE™ Manuscripts

Effects of comparing 2 liters polyethylene glycol alone or plus ascorbic acid and 4 liters polyethylene glycol alone with each other for bowel preparation before colonoscopy: protocol for a systematic review and network meta-analysis

For peer review only

Xu Tian[1,2*], Wei-Qing Chen[1*], Jie-Li Huang[1], Lan-Ying He[1], Bang-Lun Liu[1], Xi Liu[1], Hang Zhou[1], Bing-Rong Liu[3]

*These authors have contributed equally to this work as first author.

[1]Department of Gastroenterology, Chongqing Cancer Institute & Hospital & Cancer Center, Chongqing 400030, China

[2]Editorial Office, TMR Integrative Nursing, TMR Publishing Group, Tianjin 300170, China

[3]The Gastrointestinal Hospital, the First Affiliated Hospital of Zhengzhou University, Zhengzhou 450052, China

Corresponding author: Wei-Qing Chen, MD, Department of Gastroenterology, Chongqing Cancer Institute & Hospital & Cancer Center, 181 Hanyu Road, Shapingba District, Chongqing 400030, China; telephone number: (86) 0 18222275023, e-mail: chenwq20140809@163.com

For peer review only - http://bmjopen.bmj.com/site/about/guidelines.xhtml

ABSTRACT

Introduction Colonoscopy has been regarded as a standard method of early detecting and removing gastrointestinal lesions, while adequate bowel preparation is the prerequisite of determining the diagnostic accuracy and treatment safety of this process. Polyethylene glycol (PEG)-based bowel preparation regime remains the first recommendation, but optimal option is still uncertainty. The aim of this systematic review and network meta-analysis of randomized controlled trials (RCTs) is to determine the optimal PEG-based bowel preparation regime before colonoscopy.

Methods and analysis We will assign two investigators to independently search all potential citations, screen records, abstract essential information, and appraise risk of bias accordingly. And then, random effects pairwise and network meta-analyses of RCTs comparing 2 liters PEG alone or plus ascorbic acid (Asc) and 4 liters PEG alone with each other will be performed by using RevMan 5.3 (Copenhagen: The Nordic Cochrane Centre, The Cochrane Collaboration, 2013), Stata 14 (StataCorp, Texas) and WinBUGS 1.4 (imperial College School of Medicine at St Mary's, London) from January 2000 to April 2017. The surface under the cumulative ranking curve (SCURA) will also be calculated in order to rank regimes.

Ethics and dissemination The ethics approval and patient written informed consent will not be required because of all analyses in the present study will be performed based on data from published studies. We will submit our systematic review and network meta-analysis to a peer-reviewed scientific journal for publication.

Systematic review registration PROSPRO: CRD42017068957

For peer review only - http://bmjopen.bmj.com/site/about/guidelines.xhtml

For peer review only

Strengths and limitations of this study

- The protocol addresses the important question of which 2 liters PEG alone or plus Asc and 4 liters PEG alone regime offers the most benefits for bowel preparation efficacy before colonoscopy.
- The present network meta-analysis has a clearly established aim, stringent inclusion criteria, state-of-the-art methods for data collection and quantitative synthesis.
- The present network meta-analysis will design a series of established methods to increase the reliability of pooled results through rationally addressing heterogeneity and risk of bias.
- The present network meta-analysis will rank all investigated PEG-based bowel preparation regimes in terms of each outcome, which facilitates evidence-informed decision-making.
- Limitations include variations in administration times of drinking the same bowel preparation regimes, diet description prior to colonoscopy, type of colonoscopies, and assessment tool for bowel preparation efficacy.

For peer review only

For peer review only - http://bmjopen.bmj.com/site/about/guidelines.xhtml

BACKGROUND

Colorectal cancer (CRC) is one of the most common cancers diagnosed in the world and it is also the major contributor to cancer-associated morbidity and mortality [1]. Colonoscopy has been considered as the most effective method for early detection and prevention of CRC [2]. Published evidences suggested that early detection and endoscopic resection of polyps and abnormal lesions gastrointestinal tract can reduce approximately 50% mortality of CRC [3,4]. It is noted that, however, the adequate bowel preparation is the prerequisite of guaranteeing diagnostic accuracy and therapeutic safety of colonoscopy [5]. Issued data illustrated a sever fact that more than 40% of colonoscopy failures resulted from inadequate bowel preparation [6]. Moreover, inadequate bowel preparation also caused other negative consequences such as missed detection of polyps or lesions, increased risk of procedure-related complications, and increased economic costs [7]. Several factors can affect the quality of bowel preparation [8], and low patient-based compliance, poor palatability of bowel preparation solution, and inevitable requirement of drinking a large volume of preparation solution account for 20% to 25% inadequate bowel preparations [7]. However, low patient-based compliance with recommend regime play a decisive role in the overall success of the procedure [9].

For the purpose of improving quality of bowel preparation, several regimes have been developed such as polyethylene glycol (PEG)-based solutions, sodium phosphate (NaP), and sodium picosulfate solutions. Of these all regimes, PEG-based regimes are still first recommendation [10]. Several modified regimes, including split-dose regime,

For peer review only - http://bmjopen.bmj.com/site/about/guidelines.xhtml

low-volume regime, low-volume plus ascorbic acid (Asc), have been designed because patients are difficult to intake traditional 4L PEG owing to the large volume of fluid and poor palatability [11]. A series of randomized controlled trials (RCTs) have been performed to investigate the comparative efficacy of split dose versus single dose [12], low volume (2L) plus Asc versus traditional volume (4L) [13], and low volume plus Asc versus low volume [14]. However, the study regarding low volume versus traditional volume, low volume versus low volume plus Asc with split dose, and low volume versus traditional volume with split dose have not yet been identified. Moreover, individual study is difficult to identify subtle clinical differences owing to the smaller patient number [15]. Several meta-analyses have also been performed to evaluate the efficacy of low volume versus traditional volume [16], low volume versus plus Asc versus traditional volume [17], and split dose versus single dose [18,19]. Traditional meta-analysis methods, however, is unable to investigate the comparative efficacy of more than 2 interventions.

In order to solve the limitations of traditional meta-analysis technique, Bayesian network meta-analysis based on Markov Chain Monte Carlo (MCMC) and Gibbs Sampling, the expansion of pairwise meta-analysis, has been developed to evaluate the comparative efficacy of multiple treatments which are not directly compared in individual RCT [20]. And thus, we proposed this network meta-analysis to establish the effects of comparing 2 liters PEG alone or plus Asc and 4 liters PEG alone with each other prior to colonoscopy. We designed this systematic review and network meta-analysis on May 10, 2017; and we expected to complete this study on December

For peer review only - http://bmjopen.bmj.com/site/about/guidelines.xhtml

31, 2017.

METHODS AND DESIGN

We designed and completed this protocol for systematic review and network meta-analysis according to the preferred reporting items for systematic review and meta-analysis protocols (PRISMA-P) 2015: elaboration and explanation [21]. The systematic review and network meta-analysis was registered in International Prospective Register of Systematic Reviews (PROSPERO) (CRD42017068957). We will perform this traditional pairwise and network meta-analysis in accordance with the Cochrane Handbook for Systematic Reviews of Interventions [22] and report all results according to the preferred reporting items for systematic review and meta-analysis for network meta-analysis (PRISMA-NMA) [23].

Selection criteria

In our meta-analysis, the study will be considered if the following inclusion criteria are met: (i) Patients: all adult patients undergoing elective colonoscopy in endoscopy center, irrespective of outpatients and inpatients; (ii) Intervention: all PEG-based bowel preparation regimes including 4L PEG and 2L PEG plus Asc with single or split dose and did not combine with other drugs, we drawn the possible evidence network according to the targeted regimes in terms of bowel preparation efficacy (see **Figure 1**); (iii) Outcomes: bowel preparation efficacy (BPE) was regarded as primary outcome, and the secondary outcomes including compliance with recommend regime

For peer review only - http://bmjopen.bmj.com/site/about/guidelines.xhtml

(CP), preference to repeat the same regime (PRSR), acceptance to regime (AT), adverse events (AEs), and detection rate of polyps and adenomas (DRPA) and colorectal cancer (DRCRC); (iv) Study design: only RCTs were included, abstract with sufficient data was also considered; and (vi) Language: only full-text published in English- and Chinese Language will be considered because of translator who is well versed in other languages was not included.

Study will be excluded if it met at least one of following criteria: (i) essential information cannot be extracted; (ii) duplication with poor methodology and insufficient data; (iii) non-original research types such as review, editorial, letter to the editor and comments; and (iv) the study investigating the potential of bowel preparation regime in special patients such as elderly or previous poor bowel preparation.

Definition of outcomes

In our systemic review and network meta-analysis, the BPE was also regarded as successful bowel preparation, and it was defined an Ottawa score of < 5 or a Boston Bowel Preparation Scale (BBPS) score of $\geqslant 2$ for all locations or an excellent or good bowel preparation designation on the Aronchik scale or other non-validated 3-, 4-, or 5-point scales (excellent, good, fair, poor, very poor), which was rated by colonoscopist during performing colonoscopy. CP was defined as adherence to the bowel preparation prescribed or consumption of at least 75% of the prescribed bowel preparation, which was evaluated before performed the colonoscopy. PRSR, AT and

For peer review only - http://bmjopen.bmj.com/site/about/guidelines.xhtml

AEs were measured by using the specified questionnaires in each eligible study after completed the colonoscopy examine (i.e. defined by individual study). DRPA and DRCRC refer to the number of detecting actually polyps and adenomas and CRC respectively, which were all established histopathologically.

Identification of citations

We will firstly electronically search the PubMed, Cochrane Central Register of Controlled Trials (CENTRAL), EMBASE, China National Knowledge Infrastructure (CNKI), and Chinses Biomedical Literatures database (CBM) in order to capture all potential records investigating the comparative efficacy of different PEG-based bowel preparation regimes from January 2000 to April 2017. "*Colonoscopy*", "*polyethylene glycols*", and "*random*" were used to construct search algorithms in accordance with the requests of targeted databases, and all possible search algorithms have been documented in electronical supplementary material Table 1 **(ESM-Table 1)**.

After electronic search, we will also hand check the reference lists of all eligible studies and topic-related review and electronically retrieve the Clinicaltrial.gov for the purpose of covering all potential eligible study. It is noted that, however, only studies published in English- and Chinese language will be considered in our systematic review and network meta-analysis.

Data extraction

We have designed a standard data extraction form before performed our previous

For peer review only - http://bmjopen.bmj.com/site/about/guidelines.xhtml

For peer review only

two systematic reviews and network meta-analyses (see electronical supplementary material-SDE). All captured citations will be imposed into EndNote literature management software version X7. We will consequently assign two reviewers to abstract the basic information and data for specific outcome from eligible study, such as first author, publication year, age of participants, sample size, bowel preparation regimes, and outcomes of interest using this standard data extraction form [24]. We will contact the corresponding author if sufficient data of an eligible study cannot be abstracted from full-text. The Kappa value will be calculated in order to assess the inter-investigator reliability. We will establish the consensus principle to be the method of resolving divergences between reviewers.

Quality assessment of individual study

We will assign two independent reviewers to appraise the risk of bias from seven domains including randomization sequence generation, allocation concealment, blinding of participants, blinding of study personnel, blinding of outcome assessors, incomplete outcome data, selective reporting and other bias with the Cochrane risk of bias assessment tool [22,25]. A study will be assigned into a level of '*high risk of bias*', '*unclear risk of bias*' or '*low risk of bias*' according to the match level between actual information and evaluation criteria [22].

Description of the available data

We derived each pairwise comparison from descriptive statistics on available data

For peer review only - http://bmjopen.bmj.com/site/about/guidelines.xhtml

and selected variables for study and population characteristics such as age, study length and outcome-relevant baseline risk factors. A network diagram was used for each outcome to present the direct comparisons between different bowel preparation regimes and control groups. In these diagrams, nodes (circles) represented various bowel preparations and their sizes were proportional to the sample size of each respective intervention; edges (lines) indicated direct comparisons and their thicknesses were proportional to the standard error (precision).

Statistical analysis

We will firstly perform traditional pairwise meta-analysis based on random effect model, which incorporates within- and between-studies heterogeneity, to estimate the summarized odd ratio (OR) and 95% confidence intervals (CIs) [26]. Chi^2 method will be adopted to test the heterogeneity [27] and I^2 statistic will be used to estimate the proportion of the overall variation that is attributable to between-study heterogeneity [28]. The value of I^2 statistic is larger than 50% indicating substantial heterogeneity [28]. We will draw the funnel plot to identify publication bias if the number of studies analyzed was more than 10 [29]. The studies with more than two comparison groups will be quantitatively incorporated in pairwise meta-analysis according to the specific comparison.

Following the traditional pairwise meta-analysis, random-effects network meta-analysis will be performed according to the methods described by Chaimani and colleagues [30]. The initial values, automatically generated from software, will be used

For peer review only - http://bmjopen.bmj.com/site/about/guidelines.xhtml

to fit the model [31]. We plan to perform 70000 iterations and 30000 burn-in for each outcome and convergence.

The surface under the cumulative ranking curve (SUCRA) will also be drawn in order to rank all PEG-based bowel preparation regimes and a higher value suggests better results for respective regime [32].

All analyses will be conducted by using the RevMan 5.3 (Copenhagen: The Nordic Cochrane Centre, The Cochrane Collaboration, 2013), Stata 14 (StataCorp, Texas) and WinBUGS 1.4 (imperial College School of Medicine at St Mary's, London).

Assessment of small study effects and inconsistency

We will generate the comparison-adjusted funnel plot to assess the small-study effects when the number of studies included in one pair of comparison was more than 10 [33]. We will calculate the inconsistency factor based on the loop-specific method to assess the inconsistency [30].

Subgroup and sensitivity analyses

In case of possible important heterogeneity or inconsistency, we explored the possible sources using subgroup and meta-regression analyses. Subgroup analyses were planned for time of colonoscopy, patient sources and age. Sensitivity analyses were planned for bowel preparation quality by analyzing only studies considered being at low risk of bias.

For peer review only

DISCUSSION

The CRC is one of the most common malignancies, and issued statistics illustrated that it is the fourth contributor to the cancer-death worldwide [1]. Colonoscopy has been regarded as the standard process of early preventing and detecting CRC in clinical practice [2]. However, diagnostic accuracy and operation safety during performing colonoscopy mainly depend on the quality of bowel preparation [34]. Although several novel bowel preparation regimes have been developed in order to improve the tolerability and compliance of patients, the PEG-based regimes have been first-line recommendation [10]. It is noted that several modified regimes have been applied in clinical practice, but no primary study and traditional pairwise meta-analysis comparing various PEG-based bowel preparation regimes with each other has been published. And thus it is still debate which PEG-based regime should be optimally described. We hence proposed this network meta-analysis to determine the optimal PEG-based regime for the purpose of facilitating the informed-decision making.

This network meta-analysis was one of the first to compare the direct and indirect effects of different PEG-based regimes for bowel preparation prior to colonoscopy. The results of the present network meta-analysis will influence evidence-based decision-making in bowel preparation regime prescription, since it will be fundamental for reliable recommendations in the consideration of bowel preparation regime before colonoscopy.

Ethics and Dissemination

For peer review only - http://bmjopen.bmj.com/site/about/guidelines.xhtml

The ethics approval and patient written informed consent will not be required because of all analyses in the present study will be performed based on data from published studies. We will submit our systematic review and network meta-analysis to a peer-reviewed scientific journal for publication.

REFERENCES

1. Torre LA, Bray F, Siegel RL, Ferlay J, Lortet-Tieulent J, Jemal A. Global cancer statistics, 2012. *CA Cancer J Clin.* Mar 2015;65(2):87-108.
2. Lieberman DA, Weiss DG, Bond JH, Ahnen DJ, Garewal H, Chejfec G. Use of colonoscopy to screen asymptomatic adults for colorectal cancer. Veterans Affairs Cooperative Study Group 380. *The New England journal of medicine.* Jul 20 2000;343(3):162-168.
3. Quintero E, Castells A, Bujanda L, et al. Colonoscopy versus fecal immunochemical testing in colorectal-cancer screening. *The New England journal of medicine.* Feb 23 2012;366(8):697-706.
4. Zauber AG, Winawer SJ, O'Brien MJ, et al. Colonoscopic polypectomy and long-term prevention of colorectal-cancer deaths. *The New England journal of medicine.* Feb 23 2012;366(8):687-696.
5. Marmo R, Rotondano G, Riccio G, et al. Effective bowel cleansing before colonoscopy: a randomized study of split-dosage versus non-split dosage regimens of high-volume versus low-volume polyethylene glycol solutions. *Gastrointestinal endoscopy.* Aug 2010;72(2):313-320.
6. Ponchon T, Boustière C, Heresbach D, Hagege H, Tarrerias A, Halphen M. A low-volume polyethylene glycol plus ascorbate solution for bowel cleansing prior to colonoscopy: the NORMO randomised clinical trial. *Digestive and liver disease : official journal of the Italian Society of Gastroenterology and the Italian Association for the Study of the Liver.* 2013;45(10):820-826. http://onlinelibrary.wiley.com/o/cochrane/clcentral/articles/982/CN-01122982/frame.html.
7. Froehlich F, Wietlisbach V, Gonvers JJ, Burnand B, Vader JP. Impact of colonic cleansing on quality and diagnostic yield of colonoscopy: the European Panel of Appropriateness of Gastrointestinal Endoscopy European multicenter study. *Gastrointestinal endoscopy.* Mar 2005;61(3):378-384.
8. Kaminski MF, Regula J, Kraszewska E, et al. Quality indicators for colonoscopy and the risk of interval cancer. *The New England journal of medicine.* May 13 2010;362(19):1795-1803.
9. Ness RM, Manam R, Hoen H, Chalasani N. Predictors of inadequate bowel preparation for colonoscopy. *Am J Gastroenterol.* Jun 2001;96(6):1797-1802.
10. Mathus-Vliegen E, Pellise M, Heresbach D, et al. Consensus guidelines for the use of bowel preparation prior to colonic diagnostic procedures: colonoscopy and small bowel video capsule endoscopy. *Current medical research and opinion.* Aug 2013;29(8):931-945.

For peer review only

11. Ell C, Fischbach W, Bronisch HJ, et al. Randomized trial of low-volume PEG solution versus standard PEG + electrolytes for bowel cleansing before colonoscopy. *Am J Gastroenterol.* Apr 2008;103(4):883-893.

12. Kim M, Park J, Park J, et al. Does Polyethylene Glycol (PEG) Plus Ascorbic Acid Induce More Mucosal Injuries than Split-Dose 4-L PEG during Bowel Preparation? *Gut and liver.* 2017;10(2):237-243. http://onlinelibrary.wiley.com/o/cochrane/clcentral/articles/765/CN-01260765/frame.html.

13. Rivas J, Perez A, Hernandez M, Schneider A, Castro F. Efficacy of morning-only 4 liter sulfa free polyethylene glycol vs 2 liter polyethylene glycol with ascorbic acid for afternoon colonoscopy. *World journal of gastroenterology.* 2014;20(30):10620-10627. http://onlinelibrary.wiley.com/o/cochrane/clcentral/articles/049/CN-01040049/frame.html.

14. Kanie H, Kojima I, Araki H, et al. A randomized clinical trial of low-volume polyethylene glycol versus low-volume polyethylene glycol with ascorbic acid solutions. *Gastrointestinal endoscopy.* 2016;83(5):AB226.

15. Wang J, Meng XH, Guo ZM, Wu YH, Zhao JG. Interventions for treating displaced midshaft clavicular fractures: a Bayesian network meta-analysis of randomized controlled trials. *Medicine (Baltimore).* Mar 2015;94(11):e595.

16. Godfrey J, Clark R, Choudhary A, Ashraf I, Matteson M, Bechtold M. Low-volume polyethylene glycol and ascorbic acid for bowel preparation prior to colonoscopy: A meta-analysis. *American Journal of Gastroenterology.* 2011;106:S527.

17. Xie Q, Chen L, Zhao F, et al. A meta-analysis of randomized controlled trials of low-volume polyethylene glycol plus ascorbic acid versus standard-volume polyethylene glycol solution as bowel preparations for colonoscopy. *PloS one.* 2014;9(6):e99092.

18. Avalos DJ, Sarkis FS, Keihanian T, et al. Same-day morning bowel regimens vs. Split regimens for colonoscopy: A meta-analysis of randomized controlled trials. *Gastroenterology.* 2016;150(4):S839.

19. Ménard C, Barkun AN, Martel M, Restellini S, Kherad O. Split-dose versus same-day bowel preparations for colonoscopy: A meta-analysis. *Gastroenterology.* 2016;150(4):S838.

20. Mills EJ, Ioannidis JP, Thorlund K, Schunemann HJ, Puhan MA, Guyatt GH. How to use an article reporting a multiple treatment comparison meta-analysis. *Jama.* Sep 26 2012;308(12):1246-1253.

21. Shamseer L, Moher D, Clarke M, et al. Preferred reporting items for systematic review and meta-analysis protocols (PRISMA-P) 2015: elaboration and explanation. *BMJ.* Jan 02 2015;349:g7647.

22. Higgins JP, Green S. Cochrane Handbook For Systematic Reviews Of Interventions Version 5.0.0. *Naunyn-Schmiedebergs Archiv für experimentelle Pathologie und Pharmakologie.* 2009;210(2):S38.

23. Hutton B, Salanti G, Caldwell DM, et al. The PRISMA extension statement for reporting of systematic reviews incorporating network meta-analyses of health care interventions: checklist and explanations. *Ann Intern Med.* Jun 02 2015;162(11):777-784.

24. Song GM, Tian X, Zhang L, et al. Immunonutrition Support for Patients Undergoing Surgery for Gastrointestinal Malignancy: Preoperative, Postoperative, or Perioperative? A Bayesian Network Meta-Analysis of Randomized Controlled Trials. *Medicine (Baltimore).* Jul 2015;94(29):e1225.

For peer review only

For peer review only - http://bmjopen.bmj.com/site/about/guidelines.xhtml

25. Zeng X, Zhang Y, Kwong JS, et al. The methodological quality assessment tools for preclinical and clinical studies, systematic review and meta-analysis, and clinical practice guideline: a systematic review. *J Evid Based Med.* Feb 2015;8(1):2-10.

26. DerSimonian R, Laird N. Meta-analysis in clinical trials. *Controlled clinical trials.* Sep 1986;7(3):177-188.

27. Bowden J, Tierney JF, Copas AJ, Burdett S. Quantifying, displaying and accounting for heterogeneity in the meta-analysis of RCTs using standard and generalised Q statistics. *BMC medical research methodology.* 2011;11:41.

28. Higgins JPT, Thompson SG. Quantifying heterogeneity in a meta-analysis. *Statistics in Medicine.* 2002;21(11):1539-1558.

29. Tian X, Zhou JG, Zeng Z, et al. Cetuximab in patients with esophageal cancer: a systematic review and meta-analysis of randomized controlled trials. *Medical Oncology.* 2015;32(4):1-10.

30. Chaimani A, Higgins JPT, Mavridis D, Spyridonos P, Salanti G. Graphical Tools for Network Meta-Analysis in STATA. *Plos One.* 2013;8(10):e76654-e76654.

31. Sutton DA, Ades AE, Cooper N, Abrams K. Use of Indirect and Mixed Treatment Comparisons for Technology Assessment. *Pharmacoeconomics.* 2008;26(9):753-767.

32. Georgia S, Ades AE, Ioannidis JPA. Graphical methods and numerical summaries for presenting results from multiple-treatment meta-analysis: an overview and tutorial. *Journal of Clinical Epidemiology.* 2011;64(2):163-171.

33. Chen LX, Zhou ZR, Li YL, et al. Comparison of Bone Mineral Density in Lumbar Spine and Fracture Rate among Eight Drugs in Treatments of Osteoporosis in Men: A Network Meta-Analysis. *Plos One.* 2015;10(5).

34. Rapier R, Houston C. A prospective study to assess the efficacy and patient tolerance of three bowel preparations for colonoscopy. *Gastroenterology nursing : the official journal of the Society of Gastroenterology Nurses and Associates.* Jul-Aug 2006;29(4):305-308.

Contributors

X.T. and W.-Q.C. conceived and designed this study; X.T. and J.-L.H. searched and selected studies; L.-Y.H. and B.-L.L. extracted essential information; X.T. and B.-R.L. assessed the risk of bias; X.T., W.-Q.C., X.L. and H.Z. performed statistical analyses; X.T. and W.-Q.C. interpreted the pooled results; X.T., W.-Q.C. and B.-R.L. drafted the manuscript; all authors approved this manuscript to be considered for publication.

Funding statement

This research received no specific grant from any funding agency in the public,

For peer review only - http://bmjopen.bmj.com/site/about/guidelines.xhtml

commercial or not-for-profit sectors.

Competing interests

None declared.

For peer review only

For peer review only - http://bmjopen.bmj.com/site/about/guidelines.xhtml

FIGURE LENGENDS

Figure 1 Possible evidence network of all possible PEG-based bowel preparation regimes in terms of bowel preparation efficacy. The yellow solid line indicates direct comparisons between regimes which were directly compared in original studies. The brown node represents the each PEG-based bowel preparation regime. PEG = polyethylene glycol, SD = split dose, Asc = ascorbic acid.

For peer review only

For peer review only - http://bmjopen.bmj.com/site/about/guidelines.xhtml

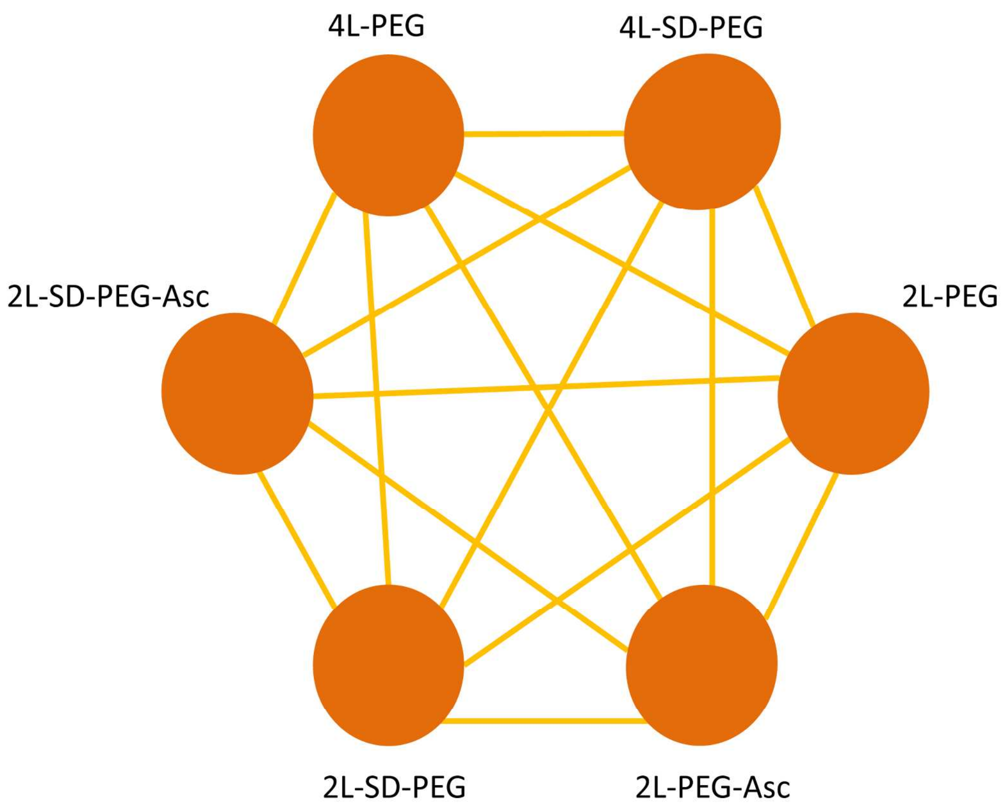

The yellow solid line indicates direct comparisons between regimes which were directly compared in original studies. The brown node represents the each PEG-based bowel preparation regime. PEG = polyethylene glycol, SD = split dose, Asc = ascorbic acid.

185x148mm (300 x 300 DPI)

CENTRAL Search Algorithm

ID	Search
#1	Polyethylene Glycol*:ti,ab,kw or Macrogol*:ti,ab,kw or Glycol, Polyethylene:ti,ab,kw or Glycols, Polyethylene:ti,ab,kw or Polyethylene Oxide*:ti,ab,kw (Word variations have been searched)
#2	Oxide, Polyethylene:ti,ab,kw or Oxides, Polyethylene:ti,ab,kw or Polyethyleneoxide*:ti,ab,kw or Polyoxyethylene*:ti,ab,kw or Tritons:ti,ab,kw (Word variations have been searched)
#3	MeSH descriptor: [Polyethylene Glycols] explode all trees
#4	#1 or #2 or #3
#5	Colonoscop*:ti,ab,kw or Colonoscopic Surgical Procedure*:ti,ab,kw or Procedure, Colonoscopic Surgical:ti,ab,kw or Procedures, Colonoscopic Surgical:ti,ab,kw or Surgical Procedure, Colonoscopic:ti,ab,kw (Word variations have been searched)
#6	Surgery, Colonoscopic:ti,ab,kw or Surgical Procedures, Colonoscopic:ti,ab,kw or Colonoscopic Surger*:ti,ab,kw or Surgeries, Colonoscopic:ti,ab,kw (Word variations have been searched)
#7	MeSH descriptor: [Colonoscopy] explode all trees
#8	#5 or #6 or #7
#9	random*:ti,ab,kw (Word variations have been searched)
#10	MeSH descriptor: [Randomized Controlled Trial] explode all trees
#11	MeSH descriptor: [Randomized Controlled Trials as Topic] explode all trees
#12	#9 or #10 or #11
#13	#4 and #8 and #12

For peer review only

PubMed Search Algorithm

Search	Query
#8	Search (((("Polyethylene Glycols"[Mesh]) OR (((((((((Polyethylene Glycol*[Title/Abstract]) OR Macrogol*[Title/Abstract]) OR Glycol, Polyethylene[Title/Abstract]) OR Glycols, Polyethylene[Title/Abstract]) OR Polyethylene Oxide*[Title/Abstract]) OR Oxide, Polyethylene[Title/Abstract]) OR Oxides, Polyethylene[Title/Abstract]) OR Polyethyleneoxide*[Title/Abstract]) OR Polyoxyethylene*[Title/Abstract]) OR Tritons[Title/Abstract]))) AND (("Colonoscopy"[Mesh]) OR (((((((((Colonoscop*[Title/Abstract]) OR Colonoscopic Surgical Procedure*[Title/Abstract]) OR Procedure, Colonoscopic Surgical[Title/Abstract]) OR Procedures, Colonoscopic Surgical[Title/Abstract]) OR Surgical Procedure, Colonoscopic[Title/Abstract]) OR Surgery, Colonoscopic[Title/Abstract]) OR Surgical Procedures, Colonoscopic[Title/Abstract]) OR Colonoscopic Surger*[Title/Abstract]) OR Surgeries, Colonoscopic[Title/Abstract]))) AND random*[Title/Abstract] Sort by: PublicationDate
#7	Search random*[Title/Abstract] Sort by: PublicationDate
#6	Search ("Colonoscopy"[Mesh]) OR (((((((((Colonoscop*[Title/Abstract]) OR Colonoscopic Surgical Procedure*[Title/Abstract]) OR Procedure, Colonoscopic Surgical[Title/Abstract]) OR Procedures, Colonoscopic Surgical[Title/Abstract]) OR Surgical Procedure, Colonoscopic[Title/Abstract]) OR Surgery, Colonoscopic[Title/Abstract]) OR Surgical Procedures, Colonoscopic[Title/Abstract]) OR Colonoscopic Surger*[Title/Abstract]) OR Surgeries, Colonoscopic[Title/Abstract]) Sort by: PublicationDate
#5	Search ((((((((Colonoscop*[Title/Abstract]) OR Colonoscopic Surgical Procedure*[Title/Abstract]) OR Procedure, Colonoscopic Surgical[Title/Abstract]) OR Procedures, Colonoscopic Surgical[Title/Abstract]) OR Surgical Procedure, Colonoscopic[Title/Abstract]) OR Surgery, Colonoscopic[Title/Abstract]) OR Surgical Procedures, Colonoscopic[Title/Abstract]) OR Colonoscopic Surger*[Title/Abstract]) OR Surgeries, Colonoscopic[Title/Abstract] Sort by: PublicationDate
#4	Search "Colonoscopy"[Mesh] Sort by: PublicationDate
#3	Search ("Polyethylene Glycols"[Mesh]) OR (((((((((Polyethylene Glycol*[Title/Abstract]) OR Macrogol*[Title/Abstract]) OR Glycol, Polyethylene[Title/Abstract]) OR Glycols, Polyethylene[Title/Abstract]) OR Polyethylene Oxide*[Title/Abstract]) OR Oxide, Polyethylene[Title/Abstract]) OR Oxides, Polyethylene[Title/Abstract]) OR Polyethyleneoxide*[Title/Abstract]) OR Polyoxyethylene*[Title/Abstract]) OR Tritons[Title/Abstract]) Sort by: PublicationDate
#2	Search ((((((((Polyethylene Glycol*[Title/Abstract]) OR Macrogol*[Title/Abstract]) OR Glycol, Polyethylene[Title/Abstract]) OR Glycols, Polyethylene[Title/Abstract]) OR Polyethylene Oxide*[Title/Abstract]) OR Oxide, Polyethylene[Title/Abstract]) OR Oxides, Polyethylene[Title/Abstract]) OR Polyethyleneoxide*[Title/Abstract]) OR Polyoxyethylene*[Title/Abstract]) OR Tritons[Title/Abstract] Sort by: PublicationDate
#1	Search "Polyethylene Glycols"[Mesh] Sort by: PublicationDate

For peer review only

Embase Search Algorithm

No. Query

#10. polyethylene:ab,ti AND glycol*:ab,ti OR macrogol*:ab,ti OR (glycol,:ab,ti AND polyethylene:ab,ti) OR (glycols,:ab,ti AND polyethylene:ab,ti) OR (polyethylene:ab,ti AND oxide*:ab,ti) OR (oxide,:ab,ti AND polyethylene:ab,ti) OR (oxides,:ab,ti AND polyethylene:ab,ti) OR polyethyleneoxide*:ab,ti OR polyoxyethylene:ab,ti OR tritons:ab,ti OR 'macrogol derivative'/exp AND (colonoscop*:ab,ti OR (colonoscopic:ab,ti AND surgical:ab,ti AND procedure*:ab,ti) OR (procedure,:ab,ti AND colonoscopic:ab,ti AND surgical:ab,ti) OR (procedures,:ab,ti AND colonoscopic:ab,ti AND surgical:ab,ti) OR (surgical:ab,ti AND procedure,:ab,ti AND colonoscopic:ab,ti) OR (surgery,:ab,ti AND colonoscopic:ab,ti) OR (surgical:ab,ti AND procedures,:ab,ti AND colonoscopic:ab,ti) OR (colonoscopic:ab,ti AND surger*:ab,ti) OR (surgeries,:ab,ti AND colonoscopic:ab,ti) OR 'colonoscopy'/exp) AND (random*:ab,ti OR 'randomized controlled trial'/exp OR 'randomized controlled trial (topic)'/exp)

#9. random*:ab,ti OR 'randomized controlled trial'/exp OR 'randomized controlled trial (topic)'/exp

#8. 'randomized controlled trial'/exp OR 'randomized controlled trial (topic)'/exp

#7. random*:ab,ti

#6. colonoscop*:ab,ti OR (colonoscopic:ab,ti AND surgical:ab,ti AND procedure*:ab,ti) OR (procedure,:ab,ti AND colonoscopic:ab,ti AND surgical:ab,ti) OR (procedures,:ab,ti AND colonoscopic:ab,ti AND surgical:ab,ti) OR (surgical:ab,ti AND procedure,:ab,ti AND colonoscopic:ab,ti) OR (surgery,:ab,ti AND colonoscopic:ab,ti) OR (surgical:ab,ti AND procedures,:ab,ti AND colonoscopic:ab,ti) OR (colonoscopic:ab,ti AND surger*:ab,ti) OR (surgeries,:ab,ti AND colonoscopic:ab,ti) OR 'colonoscopy'/exp

#5. 'colonoscopy'/exp

#4. colonoscop*:ab,ti OR (colonoscopic:ab,ti AND surgical:ab,ti AND procedure*:ab,ti) OR (procedure,:ab,ti AND colonoscopic:ab,ti AND surgical:ab,ti) OR (procedures,:ab,ti AND colonoscopic:ab,ti AND surgical:ab,ti) OR (surgical:ab,ti AND procedure,:ab,ti AND colonoscopic:ab,ti) OR (surgery,:ab,ti AND colonoscopic:ab,ti) OR (surgical:ab,ti AND

For peer review only

procedures,:ab,ti AND colonoscopic:ab,ti) OR (colonoscopic:ab,ti AND surger*:ab,ti) OR (surgeries,:ab,ti AND colonoscopic:ab,ti)

#3. polyethylene:ab,ti AND glycol*:ab,ti OR macrogol*:ab,ti OR (glycol,:ab,ti AND polyethylene:ab,ti) OR (glycols,:ab,ti AND polyethylene:ab,ti) OR (polyethylene:ab,ti AND oxide*:ab,ti) OR (oxide,:ab,ti AND polyethylene:ab,ti) OR (oxides,:ab,ti AND polyethylene:ab,ti) OR polyethyleneoxide*:ab,ti OR polyoxyethylene:ab,ti OR tritons:ab,ti OR 'macrogol derivative'/exp

#2. 'macrogol derivative'/exp

#1. polyethylene:ab,ti AND glycol*:ab,ti OR macrogol*:ab,ti OR (glycol,:ab,ti AND polyethylene:ab,ti) OR (glycols,:ab,ti AND polyethylene:ab,ti) OR (polyethylene:ab,ti AND oxide*:ab,ti) OR (oxide,:ab,ti AND polyethylene:ab,ti) OR (oxides,:ab,ti AND polyethylene:ab,ti) OR polyethyleneoxide*:ab,ti OR polyoxyethylene:ab,ti OR tritons:ab,ti

For peer review only

<table>
<tr><th colspan="5">Basic Characteristics of Included into Study</th></tr>
<tr><td colspan="2" rowspan="2">Study Information</td><td>Author</td><td>Year</td><td>Country</td></tr>
<tr><td></td><td></td><td></td></tr>
<tr><td rowspan="4">Methods</td><td>Allocation</td><td colspan="3"></td></tr>
<tr><td>Duration</td><td colspan="3"></td></tr>
<tr><td>Blinding</td><td colspan="3"></td></tr>
<tr><td>Location</td><td colspan="3"></td></tr>
<tr><td rowspan="9">Participants</td><td>Diagnosis</td><td colspan="3">Pts were diagnosed as GC based histology.</td></tr>
<tr><td rowspan="2">Age</td><td>Study Group</td><td colspan="2">Control Group</td></tr>
<tr><td></td><td colspan="2"></td></tr>
<tr><td rowspan="2">Sex</td><td>Study Group</td><td colspan="2">Control Group</td></tr>
<tr><td></td><td colspan="2"></td></tr>
<tr><td rowspan="2">Length of Illness</td><td>Study Group</td><td colspan="2">Control Group</td></tr>
<tr><td></td><td colspan="2"></td></tr>
<tr><td>Inclusion criteria</td><td colspan="3"></td></tr>
<tr><td>Exclusion criteria</td><td colspan="3"></td></tr>
<tr><td rowspan="2">Interventions</td><td>Treatment Group</td><td>Content</td><td colspan="2"></td></tr>
<tr><td>Control group</td><td>Content</td><td colspan="2"></td></tr>
<tr><td>Outcomes</td><td colspan="4"></td></tr>
<tr><td>Notes</td><td colspan="4"></td></tr>
</table>

Drop-outs	Drop out due to	Study group	Control group
	the numbers of patients in the early stage		
	the numbers of patients in the late stage		

Binary Data			
Outcomes	Name of Outcome	Event number	Total number
	Study group		
	Control group		

Binary Data			
Outcomes	Name of Outcome	Event number	Total number
	Study group		
	Control group		

Binary Data			
Outcomes	Name of Outcome	Event number	Total number
	Study group		
	Control group		

Continuous Data				
Outcomes	Name of outcome	Data extraction		
		Median	Range	P
	ECT group			
	Paroxetin group			

Continuous Data				
Outcomes	Name of outcome	Data extraction		
		Median	Range	P
	ECT group			
	Paroxetin group			

Assessing of Risk of Bias Tool		
**Item**	**Description**	**Risk of Bias**
**Sequence Generation**	Describe the method used to generate the allocation sequence in sufficient detail to allow an assessment of whether it should produce comparable groups	Was the allocation sequence adequately generated?
	Comment:	Low Risk
**Allocation Concealment**	Describe the method used to conceal the allocation sequence in sufficient detail to determine whether intervention allocations could have been foreseen	Was the allocation adequately concealed?
	Comment:	Low Risk
**Blinding of Participants and Personnel**	Describe all measures used, if any, to blind study participants and personnel from knowledge of which intervention a participant received. Provide any information relating to whether the intended blinding was effective	Was knowledge of the allocated intervention adequately prevented during the study?
	Comment:	Low Risk
**Blinding of Outcome Assessors**	Describe all measures used, if any, to blind outcome assessors from knowledge of which intervention a participant received. Provide any information relating to whether the intended blinding was effective.	Was knowledge of the allocated intervention adequately prevented during the study?
	Comment:	Unclear
**Incomplete Outcome Data**	Describe the completeness of outcome data for each main outcome, including attrition and exclusions from the analysis. State whether attrition and exclusions were reported, the numbers in each intervention group (compared with total randomized participants), reasons for attrition/exclusions where reported, and	Were incomplete outcome data adequately addressed?
	Comment:	Low Risk
**Selective Outcome Reporting**	State how the possibility of selective outcome reporting was examined by the review authors and what was found.	Are reports of the study free of suggestion of selective
	Comment:	Low Risk
**Other Bias**	State any important concerns about bias not addressed in the other domains in the tool. If particular questions/entries were re-specified in the review protocol, responses should be provided for each question/entry	Was the study apparently free of other problems that could put it at high risk of bias?
	Comment:	Low Risk

For peer review only

PRISMA-P (Preferred Reporting Items for Systematic review and Meta-Analysis Protocols) 2015 checklist: recommended items to address in a systematic review protocol*

Section and topic	Item No	Checklist item	Page
**ADMINISTRATIVE INFORMATION**			
Title:			
Identification	1a	Identify the report as a protocol of a systematic review	1
Update	1b	If the protocol is for an update of a previous systematic review, identify as such	n.a.
Registration	2	If registered, provide the name of the registry (such as PROSPERO) and registration number	n.a.
Authors:			
Contact	3a	Provide name, institutional affiliation, e-mail address of all protocol authors; provide physical mailing address of corresponding author	1
Contributions	3b	Describe contributions of protocol authors and identify the guarantor of the review	10,11
Amendments	4	If the protocol represents an amendment of a previously completed or published protocol, identify as such and list changes; otherwise, state plan for documenting important protocol amendments	n.a.
Support:			
Sources	5a	Indicate sources of financial or other support for the review	11
Sponsor	5b	Provide name for the review funder and/or sponsor	n.a.
Role of sponsor or funder	5c	Describe roles of funder(s), sponsor(s), and/or institution(s), if any, in developing the protocol	n.a.
**INTRODUCTION**			
Rationale	6	Describe the rationale for the review in the context of what is already known	3,4
Objectives	7	Provide an explicit statement of the question(s) the review will address with reference to participants, interventions, comparators, and outcomes (PICO)	4
**METHODS**			
Eligibility criteria	8	Specify the study characteristics (such as PICO, study design, setting, time frame) and report characteristics (such as years considered, language, publication status) to be used as criteria for eligibility for the review	5,6
Information sources	9	Describe all intended information sources (such as electronic databases, contact with study authors, trial registers or other grey literature sources) with planned dates of coverage	6
Search strategy	10	Present draft of search strategy to be used for at least one electronic database, including planned limits, such that it could be repeated	6, Table 1
Study records:			

For peer review only

Data management	11a	Describe the mechanism(s) that will be used to manage records and data throughout the review	7
Selection process	11b	State the process that will be used for selecting studies (such as two independent reviewers) through each phase of the review (that is, screening, eligibility and inclusion in meta-analysis)	7
Data collection process	11c	Describe planned method of extracting data from reports (such as piloting forms, done independently, in duplicate), any processes for obtaining and confirming data from investigators	7
Data items	12	List and define all variables for which data will be sought (such as PICO items, funding sources), any pre-planned data assumptions and simplifications	7
Outcomes and prioritization	13	List and define all outcomes for which data will be sought, including prioritization of main and additional outcomes, with rationale	6
Risk of bias in individual studies	14	Describe anticipated methods for assessing risk of bias of individual studies, including whether this will be done at the outcome or study level, or both; state how this information will be used in data synthesis	7
Data synthesis	15a	Describe criteria under which study data will be quantitatively synthesised	8
	15b	If data are appropriate for quantitative synthesis, describe planned summary measures, methods of handling data and methods of combining data from studies, including any planned exploration of consistency (such as I^2, Kendall's τ)	8
	15c	Describe any proposed additional analyses (such as sensitivity or subgroup analyses, meta-regression)	8
	15d	If quantitative synthesis is not appropriate, describe the type of summary planned	8
Meta-bias(es)	16	Specify any planned assessment of meta-bias(es) (such as publication bias across studies, selective reporting within studies)	8
Confidence in cumulative evidence	17	Describe how the strength of the body of evidence will be assessed (such as GRADE)	11

*** It is strongly recommended that this checklist be read in conjunction with the PRISMA-P Explanation and Elaboration (cite when available) for important clarification on the items. Amendments to a review protocol should be tracked and dated.** **The copyright for PRISMA-P (including checklist) is held by the PRISMA-P Group and is distributed under a Creative Commons Attribution Licence 4.0.**

From: Shamseer L, Moher D, Clarke M, Ghersi D, Liberati A, Petticrew M, Shekelle P, Stewart L, PRISMA-P Group. Preferred reporting items for systematic review and meta-analysis protocols (PRISMA-P) 2015: elaboration and explanation. BMJ. 2015 Jan 2;349(jan02 1):g7647.

For peer review only
